# Review on modelling of thermoset and thermoplastic composites: constitutive equations, processing modelling and multiphysics modelling

**DOI:** 10.1007/s44347-026-00071-7

**Published:** 2026-11-05

**Authors:** Bhishm Dewangan, Mohammed Sirhan Pullambalavan, Andreas Chrysanthou, Tom De Vuyst, Yiding Liu

**Affiliations:** School of Physics, Engineering and Computer Science, University of Hertfordshire, Hatfield, AL10 9AB UK

**Keywords:** Thermoset composites, Thermoplastic composites, Process modelling, Multiscale modelling, Multiphysics modelling, Residual stress

## Abstract

Fibre-reinforced composites have emerged as pivotal materials in advanced structural applications across aerospace, automotive, marine, and renewable energy sectors, due to their superior specific strength, stiffness, toughness, recyclability, and rapid manufacturing potential. Composites manufacturing involves complex multiscale, multiphysics phenomena, including melting, crystallisation, flow, fibre orientation, and residual stress generation that significantly influence the final component quality and performance. Process modelling has become an indispensable tool to address these challenges, enabling the prediction of manufacturing-induced defects, residual stresses and dimensional distortion, and reduction of reliance on physical trials. This review examines current process modelling approaches for thermoset and thermoplastic fibrereinforced composites, with emphasis on the constitutive equations, process simulation methodologies and multiscale modelling frameworks used to predict process-induced phenomena. Particular attention is given to predictive frameworks for temperature evolution, crystallinity development, fibre orientation, residual stress generation, and process-induced distortion. Modelling approaches ranging from conventional finite element methods and molecular dynamics to meshless methods and machine-learning-based techniques are critically reviewed and compared with respect to their capabilities, limitations and application domains. Emphasis is given to the integration of process simulation with experimental monitoring and to the transfer of process-induced material states and defects to structural-level performance models. Key challenges that have been identified include uncertainty quantification, computational efficiency, model transferability, and the limited integration of physics-based and data-driven approaches. Future developments are expected to focus on validated multiscale process-structure-performance frameworks, uncertainty quantification, hybrid physics-informed data-driven modelling, and digital-twin-assisted process monitoring to enhance predictive reliability and accelerate the industrial adoption of composite process modelling and simulation.

## Introduction

Fibre-reinforced composites have found wide use in advanced structural applications in sectors including aerospace, marine, automotive, and renewable energy, primarily due to their exceptional stiffness and strength-to-weight ratios. Even though their production costs are typically higher than those of conventional metallic systems, the ability to minimise post-processing steps and integrate complex geometries has driven their widespread adoption. Their anisotropic nature further enables tailored mechanical performance for specific load conditions [1, 2]. Among the diverse classes of composites, thermosetting and thermoplastic composites stand out as the most widely used variants, each distinguished by unique chemical, physical, and mechanical properties that dictate their performance in different service environments. Thermosetting composites, characterised by irreversible cross-linking during curing, offer excellent dimensional stability and heat resistance, making them ideal for various applications such as aerospace structures and industrial machinery. In contrast, thermoplastic composites exhibit reversible melting and crystallisation behaviour, enabling reprocessing, recyclability, and rapid manufacturing, which renders them suitable for mass-produced components in the automotive and consumer goods sectors. Despite these significant advantages, achieving precise dimensional accuracy in composite components remains a critical and persistent challenge in their manufacturing and application.

Curing and crystallisation are fundamentally different processes, and this has direct consequences for how composite materials are modelled. Curing is an irreversible chemical reaction confined to a narrow processing window bounded by gelation, the point beyond which the resin can no longer flow, and vitrification, the point beyond which the reaction rate becomes diffusion-limited [3–5]. Once cured, the thermoset material cannot revert to its uncured state during any subsequent thermal cycle. Crystallisation, on the other hand, is a reversible physical transformation driven by nucleation and spherulitic growth on cooling, and it is governed by the temperature and cooling rates rather than a fixed reaction pathway [6–8]. This difference carries directly over to the state variables that each model family has to track. Thermoset models carry a single, monotonically increasing degree of cure, α, whereas thermoplastic models must track crystallinity as a variable that depends on the entire thermal history rather than a fixed endpoint, since the cooling rate alone can shift the level of final crystallinity by several percentage points for the same material [9]. During manufacturing, complex interactions between thermal gradients, chemical shrinkage, and mechanical constraints generate residual stresses within the material. These stresses, in turn, lead to distortions that cause discrepancies between the manufactured part and the intended mould geometry. Such deviations often manifesting as angular changes in curved components or out-of-plane deformations in flat laminates, pose significant difficulties during assembly [10, 11]. In high-precision applications, particularly in aerospace structures, even slight geometric mismatches may necessitate corrective interventions such as shimming, thereby increasing labour demands and extending production timelines. Industrial practice often relies on empirical adjustments through iterative manufacturing cycles, which are inefficient and costly for large-scale structures. Moreover, the sensitivity of distortion to multiple parameters, including stacking sequence, material characteristics, curing conditions, and tooling designs, makes the problem highly complex. Several industrial composite components including the 5 MW 27 m long A350 XWB rear wing spar, aileron ribs, fuselage stringers, and flaperons present typical examples of manufacturing-induced challenges [12, 13]. These structures are commonly produced using autoclave processing, while alternative methods such as pultrusion or resin transfer moulding may be applied depending on geometry and production volume [14]. Owing to their load critical and safety relevant roles, stringent control of manufacturing distortions and reliability is essential. Therefore, the ability to accurately predict process-induced deformation is crucial for enhancing manufacturing efficiency and ensuring the structural integrity of composite components [15–17].

Given the strong coupling between material behaviour, processing conditions and final component quality, experimental trial-and-error approaches alone are often insufficient for efficient process development and optimisation. Computational modelling of composite processing provides a powerful means to predict material behaviour during processing and identify critical regions that are prone to defect formation or distortion. Achieving this, however, requires advanced computational frameworks capable of capturing the complex, coupled mechanisms governing process-induced phenomena, including fibre-matrix interface interactions, curing kinetics (for thermosets) and crystallisation behaviour (for thermoplastics) [18]. Central to such modelling efforts is a clear understanding of the development of residual stress during manufacturing. These stresses can be broadly classified based on their scale of origin and underlying mechanisms. At the microscale, residual stresses arise within the fibre-matrix system due to mismatches in thermal expansion, resin shrinkage during curing, and environmental effects such as moisture uptake [19]. While these stresses are self-equilibrating and do not contribute significantly to global deformation, they can degrade material integrity through mechanisms such as matrix cracking. In contrast, macroscale residual stresses are primarily responsible for dimensional instability and part distortion. These stresses originate from ply-level anisotropy, inter-ply constraints, and interactions with the tooling surface [20, 21]. Furthermore, residual stresses may be categorised as thermoelastic or non-thermoelastic. Thermoelastic stresses are reversible and stem from differences between in-plane and through-thickness thermal strains, whereas non-thermoelastic stresses are irreversible and arise from more complex mechanisms, including tool–part interaction, chemical shrinkage, consolidation effects, cure or crystallinity gradients, and variations in fibre volume fraction [22]. Understanding and accurately modelling these mechanisms is essential for improving predictive capabilities and optimising composite manufacturing processes. Because several of these mechanisms act simultaneously during manufacturing, accurately predicting the final residual stress state requires coupled thermal, chemical and mechanical analyses. The relative importance of each mechanism depends on the material system, component geometry, processing route and tooling conditions.

The mechanical modelling of process-induced residual stress and shape distortion in thermoset and thermoplastic composites has been previously reviewed by Baran, Cinar [10]. However that review did not address issues related to intimate contact, bonding, void growth and polymer degradation, as these were stated to be outside its scope. Furthermore, no recent review has comprehensively integrated constitutive modelling, process simulation methodologies, multiscale approaches, machine-learning techniques, and process-structure-performance linkages within a single framework. The present review covers the same residual-stress and distortion content but also includes previously omitted aspects of manufacturing, including process steps as well as draping/forming and infusion modelling, alongside thermal, cure and crystallisation modelling. Furthermore, significant advances have been made in thermoplastic process modelling, multiscale simulation, molecular-scale modelling, machine-learning-assisted process prediction and digital-twin concepts since the earlier reviews were published. An updated assessment is therefore required to capture the emerging modelling strategies and to critically evaluate how they can be integrated within process-structure-property prediction frameworks.

**Fig. 1:**
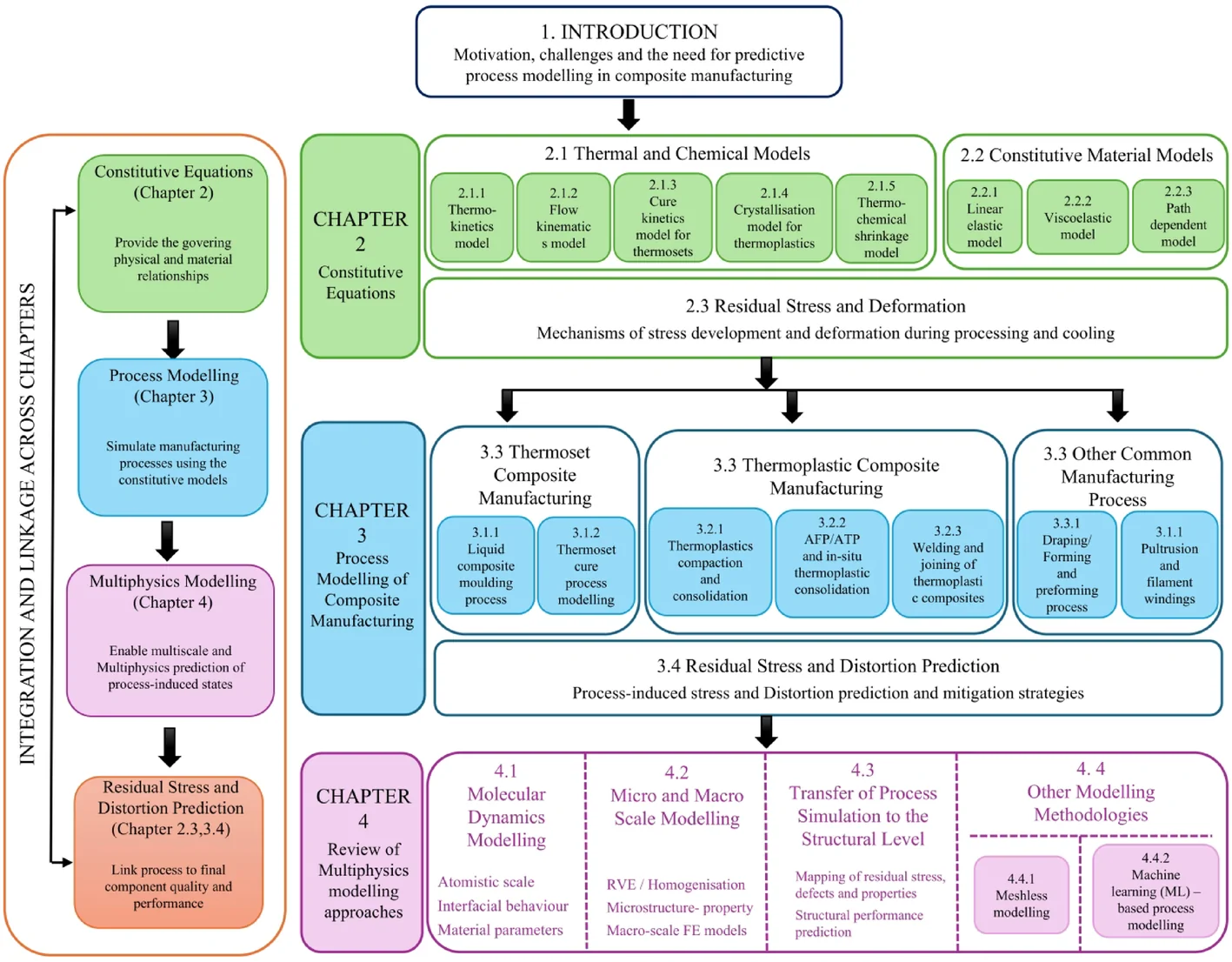
Schematic framework of this review article

This review aims to provide a critical overview of current modelling approaches for thermoset and thermoplastic composite processing, with emphasis on constitutive modelling, process simulation and multiscale process-structure-property prediction. The specific objectives of this review are to: (i) review constitutive equations governing composite processing, (ii) assess current process-simulation methodologies, (iii) evaluate multiscale and multiphysics modelling strategies, and (iv) examine the transfer of process-induced states to structural-performance predictions. The current process modelling approaches are critically examined, including Molecular Dynamics (MD), Finite Element Modelling (FEM), Meshless Modelling, Machine Learning (ML)-based modelling). This review also highlights key applications of mechanical process modelling in both thermoset and thermoplastic fibre-reinforced composites. Consideration is also given to predictive frameworks for temperature evolution, degree of cure or crystallinity, residual stress generation, and distortion during manufacturing. Although phenomena, such as intimate contact, interfacial bonding, void evolution and polymer degradation, are discussed where relevant, the primary focus of this review concerns the flow, cure, crystallisation and residual-stress modelling, while manufacturing routes such as High Pressure Resin Transfer Moulding (HP-RTM), compression RTM, thermoforming, and over-moulding are outside the scope of the present review. Figure 1 provides a schematic overview of the structure and scope of this review article, which is organised as follows. Section  2 reviews the constitutive equations governing thermal, chemical, flow and mechanical behaviour during composite processing. Section  3 examines modelling approaches for major manufacturing routes. Section  4 discusses multiscale and multiphysics methods, including molecular dynamics, homogenisation, meshless approaches and machine learning. Finally, key challenges and future research opportunities are highlighted.

## Review of the constitutive equations

### Thermal and chemical models

#### Thermo-kinetics model

Transient heat transfer plays a critical role in composite processing, as it generates temperature gradients that can lead to non-uniform vitrification or solidification across the material. Even though similar modelling approaches apply to both thermosets and thermoplastics, the heat-generation mechanisms differ; exothermic curing is the means of heat -generation in thermosets and crystallisation in thermoplastics. Since polymer transformation is strongly temperature-dependent, accurate thermal modelling is essential to predict temperature evolution during processing. According to Fourier’s law of heat conduction and the law of conservation of energy, a governing equation including both the interior source of heat$\:(\dot{\mathrm{q}})\:$and externally applied heat flux can be expressed as [23–25] (Eq. 1)

1 $$\:{\uprho\:}{\mathrm{C}}_{\mathrm{p}}\frac{\partial\:{\uptheta\:}}{\partial\:\mathrm{t}}=\:\frac{\partial\:}{\partial\:{\mathrm{x}}_{\mathrm{i}}}\left({\mathrm{k}}_{i}\frac{\partial\:{\uptheta\:}}{\partial\:{\mathrm{x}}_{\mathrm{i}}}\right)+\dot{\mathrm{q}}$$

where k represents the thermal conductivities in the i^th^ direction (in this case, it is a three-dimensional heat flow, hence, i = 1, 2, and 3), ρ represents the cured part density, and C_p_ represents the material’s specific heat. The internal heat generation ($\:\dot{\mathrm{q}})$ in the polymer matrix due to the exothermic reaction during processing, can be expressed as [10, 26, 27] (Eq. 2)

2 $$\:\dot{\mathrm{q}}={(1-{\mathrm{V}}_{\mathrm{f}}){\uprho\:}}_{\mathrm{r}}{\mathrm{H}}_{\mathrm{r}}{\mathrm{R}}_{\mathrm{r}}$$

In this equation, $\:{{\uprho\:}}_{\mathrm{r}}$ represents the density of resin, H_r_ is the total heat of reaction, V_f_ is the fibre volume fraction, and R_r_ is the reaction of curing or crystallisation as a function of temperature and degree of crystallisation of curing. Although Eq. 1 and Eq. 2 form the basis of most thermal process simulations, their predictive accuracy depends strongly on the availability of reliable temperature-dependent thermal properties and reaction kinetics data. Furthermore, in thick composite structures, strong thermal gradients and internally generated heat may lead to local temperature overshoots that cannot be captured accurately without fully coupled thermochemical analyses.

#### Flow kinematics model

The flow of resin through fibrous reinforcement media is commonly governed by Darcy’s law and can be described as (Eq. 3):

3 $$\:{\mathrm{q}}_{\mathrm{x}}=\:-\frac{\mathrm{K}}{{\upmu\:}}\frac{\mathrm{d}\mathrm{P}}{\mathrm{d}\mathrm{x}}$$

where q_x_ denotes the volumetrically averaged flow velocity, µ is the dynamic viscosity of the fluid, K represents the preform permeability, and P corresponds to the local fluid pressure. Applying mass conservation to both the solid and fluid phases yields, assuming both resin and fibres to be incompressible, can be represented as (Eq. 4):

4 $$\:-\frac{\partial\:\left({\mathrm{q}}_{\mathrm{x}}\mathrm{h}\right)}{\partial\:\mathrm{x}}=\:\frac{\partial\:\mathrm{h}}{\partial\:\mathrm{t}}$$

Here, h represents the local laminate thickness. Combining Eq. 3 and Eq. 4 can yield the governing equation (Eq. 5):

5 $$\:\frac{\partial\:}{\partial\:\mathrm{x}}\left(\frac{\mathrm{K}}{{\upmu\:}}\mathrm{h}\frac{\partial\:\mathrm{P}}{\partial\:\mathrm{x}}\right)=\:\frac{\partial\:\mathrm{h}}{\partial\:\mathrm{t}}$$

Employing a non-mixed conservative element approach [28], the flow front velocity q_x_ can be estimated as follows. Since linear finite elements yield a constant pressure gradient within each element, a Taylor series expansion around the centre of the element gives (Eq. 6):

6 $$\:{\mathrm{q}}_{\mathrm{x}}=\:-\frac{\mathrm{K}}{{\upmu\:}}\frac{\partial\:\mathrm{P}}{\partial\:\mathrm{x}}+(\mathrm{x}-{\mathrm{x}}_{\mathrm{c}})\frac{\partial\:{\mathrm{q}}_{\mathrm{x}}}{\partial\:\mathrm{x}}$$

Since both thickness and permeability vary along the spatial domain, the velocity gradient and local velocity can be expressed as (Eq. 7 and Eq. 8):

7 $$\:\:\frac{\partial\:{\mathrm{q}}_{\mathrm{x}}}{\partial\:\mathrm{x}}=\frac{\partial\:}{\partial\:\mathrm{x}}\:\left(-\frac{\mathrm{K}}{{\upmu\:}}\frac{\partial\:\mathrm{P}}{\partial\:\mathrm{x}}\right)=-\frac{1}{{\upmu\:}}\left(\frac{\partial\:\mathrm{K}}{\partial\:\mathrm{x}}\frac{\partial\:\mathrm{P}}{\partial\:\mathrm{x}}+\mathrm{K}\frac{{\partial\:}^{2}\mathrm{P}}{\partial\:{\mathrm{x}}^{2}}\right)$$

8 $$\:{\mathrm{q}}_{\mathrm{x}}=\:-\frac{\mathrm{K}}{{\upmu\:}}\frac{\partial\:\mathrm{P}}{\partial\:\mathrm{x}}+\left(\frac{\mathrm{x}-{\mathrm{x}}_{\mathrm{c}}}{\mathrm{h}}\right)\left(\frac{\mathrm{K}}{{\upmu\:}}\frac{\partial\:\mathrm{P}}{\partial\:\mathrm{x}}-\frac{\partial\:\mathrm{h}}{\partial\:\mathrm{t}}\right)$$

Darcy-based flow formulations remain the most widely adopted approach for RTM and Vacuum-Assisted Resin Transfer Moulding (VARTM) process simulations because of their simplicity and computational efficiency. However, their accuracy depends heavily on permeability characterisation, and they become less reliable when dual-scale flow, race-tracking, fibre deformation, resin bleeding, or permeability evolution during compaction significantly influence the flow behaviour.

#### Cure kinetics model for the thermoset

During resin curing, exothermic chemical reactions release substantial heat. Differential scanning calorimetry (DSC) can be employed to determine the polymerisation kinetics. The degree of curing can be measured based on the DSC is represented by [4] (Eq. 9)

9 $$\:{\upalpha\:}\left(\mathrm{t}\right)=\:\frac{\mathrm{H}\left(\mathrm{t}\right)}{{\mathrm{H}}_{\mathrm{r}}}$$

where, $\:{\upalpha\:}\left(\mathrm{t}\right)$ and H(t) denote the degree of cure and heat generated per unit mass at time t, respectively, and the total heat generated per unit mass over time is $\:{\mathrm{H}}_{\mathrm{r}}$. Here, $\:{\upalpha\:}\left(\mathrm{t}\right)$ varies from uncured stage ($\:{\upalpha\:}$ = 0) to fully cured stage ($\:{\upalpha\:}$ = 1). The differential form of curing reaction rate is described as a function of temperature (θ), and the degree of cure (α), as follows [3] (Eq. 10)

10 $$\:{\mathrm{R}}_{\mathrm{r}}=\:\frac{\mathrm{d}{\upalpha\:}}{\mathrm{d}\mathrm{t}}=\mathrm{f}({\uptheta\:},{\upalpha\:})$$

where $\:\mathrm{f}\left({\uptheta\:},{\upalpha\:}\right)\ge\:0.\:$In terms of the cure reaction, a model of cure kinetics was established by a rate Eq [5]. The cure kinetics model for epoxy system is represented by [23] (Eq. 11)

11 $$\:{\mathrm{R}}_{\mathrm{r}}=\frac{\mathrm{d}{\upalpha\:}}{\mathrm{d}\mathrm{t}}=\left({\mathrm{k}}_{1}+{\mathrm{k}}_{2}\:{{\upalpha\:}}^{\mathrm{m}}\right){\left(1-{\upalpha\:}\right)}^{\mathrm{n}}$$

where, m and n represents the exponents, k_1_ and k_2_ are described by the Arrhenius rate equations as [23] (Eq. 12)

12 $$\:{\mathrm{k}}_{1}={\mathrm{A}}_{1}\:{\mathrm{e}}^{(-\frac{\varDelta\:{\mathrm{E}}_{1}}{\mathrm{R}\mathrm{T}})}\:\:\mathrm{a}\mathrm{n}\mathrm{d}\:\:{\mathrm{k}}_{2}={\mathrm{A}}_{2}\:{\mathrm{e}}^{(-\frac{\varDelta\:{\mathrm{E}}_{2}}{\mathrm{R}\mathrm{T}})}$$

where A_1_ and A_2_ represent the pre-exponential coefficients, R is the universal gas constant in SI unit (8.314 J * ·* mol^− 1^ $\cdot\;$K^− 1^), ΔE_1_ and ΔE_2_ are the activation energies, and T is the absolute temperature in Kelvin. The Arrhenius rate in Eq. 12 assumes reaction rate governed purely by local temperature, which holds when the part conducts heat fast enough to stay close to the applied cure cycle. In thick laminates this assumption fails, because the exothermic heat release from the cure reaction itself can raise the internal temperature above the applied cure cycle, a phenomenon known as thermal overshoot or runaway [23]. Recent analytical work derives a critical thickness, for a given heating rate and resin exotherm, above which this overshoot becomes significant, thereby providing a design criterion rather than relying purely on trial-and-error cure cycle development [29, 30]. As non-uniform through-thickness cure drives non-uniform modulus development and, in turn, the residual stresses discussed in Sect. 2.2.1, 2.2.2 and 2.2.3, a single Arrhenius cure model is insufficient alone; it must be coupled with a full thermochemical FE solve.

The glass transition temperature (T_g_) is one of the key material parameters that characterise the transition of the polymer matrix from a compliant, rubber-like state to a rigid, glassy state. Its evolution during curing is commonly described as a function of the degree of cure ($\:{\upalpha\:}$). One of the most widely used relationships for this purpose is the Di Benedetto Eqs [31, 32]., which correlates T_g_ and ($\:{\upalpha\:}$) through a semi-empirical formulation given by (Eq. 13)

13 $$\:\frac{{\mathrm{T}}_{\mathrm{g}}-{\mathrm{T}}_{{\mathrm{g}}^{0}}}{{\mathrm{T}}_{{\mathrm{g}}^{{\infty\:}}}-{\mathrm{T}}_{{\mathrm{g}}^{0}}}=\:\frac{{\uplambda\:}{\upalpha\:}}{1-(1-{\uplambda\:}){\upalpha\:}}$$

In this expression $\:{\mathrm{T}}_{{\mathrm{g}}^{0}}$ and $\:{\mathrm{T}}_{{\mathrm{g}}^{{\infty\:}}}$ denote the glass transition temperatures of the uncured and fully cured resin, respectively, while $\:{\uplambda\:}$ is a dimensionless fitting parameter that governs the curvature of the transition.

Alternatively, the dependence of T_g_ on the degree of cure can be obtained directly from experimental measurements. In such cases, an empirical relationship is often adopted to represent the variation of T_g_ with $\:{\upalpha\:}$ given by [33, 34] (Eq. 14):

14 $$\:{\mathrm{T}}_{\mathrm{g}}={\mathrm{T}}_{{\mathrm{g}}^{0}}+\:{\upalpha\:}\:{\mathrm{k}}_{{\mathrm{T}}_{\mathrm{g}}}$$

where $\:{\mathrm{T}}_{{\mathrm{g}}^{0}}$ represents the glass transition temperature at a degree of cure equal to 0 and $\:{\mathrm{k}}_{{\mathrm{T}}_{\mathrm{g}}}\:$is a material-specific constant.

The rheological response of a thermosetting resin plays a critical role in governing its flow behaviour throughout processing, as it directly influences viscosity evolution. The viscosity ($\:{\upeta\:}$) of the resin is generally expressed as a function of temperature and degree of cure ($\:{\upalpha\:}$) by [10, 35](Eq. 15):

15 $$\:\:{\upeta\:}={{\upeta\:}}_{{\infty\:}}\:\mathrm{e}\mathrm{x}\mathrm{p}\left(\frac{\varDelta\:{\mathrm{E}}_{{\upeta\:}}}{\mathrm{R}\mathrm{T}}+\mathrm{K}{\upalpha\:}\right)\:\:$$

where $\:\varDelta\:{\mathrm{E}}_{{\upeta\:}}$ is the activation energy, $\:{{\upeta\:}}_{{\infty\:}}\:$is the initial viscosity, K is a material constant, R is the universal gas constant, and T is the absolute temperature. Equation 15 uses an Arrhenius-with-cure viscosity form, which couples a temperature-dependent pre-exponential term with a separately fitted function of degree of cure. The Castro-Macosko model [36] is the most widely used industrial extension, describing the divergence of viscosity with α as the resin approaches the gel point. However, chaining a separately calibrated viscosity model to the cure kinetics model accumulates error: inaccuracies in the predicted α propagate into the viscosity prediction and compound that model’s own fitting error. Newer chemo-rheological approaches address this directly by fitting viscosity as a function of processing time and temperature alone, skipping the intermediate cure kinetics step entirely and reducing compounded error, and this shown to track viscosity evolution across a broader range of industrial cure cycles than the chained model [37].

#### Crystallisation model for thermoplastics

When a thermoplastic polymer cools from the melt, its disordered structure can partially reorganise into an ordered crystalline phase, a process known as crystallisation. This occurs between the glass transition temperature and the melting point. Due to high viscosity and long molecular chains, most thermoplastics form either amorphous or semi-crystalline structures rather than fully crystalline solids. In process modelling, the cooling stage is crucial, as stiffness develops and residual stresses are generated during solidification. This differs from thermosets, where curing mainly occurs during heating [10, 35]. Most crystallisation models are based on Avrami theory [6–8], which assumes nucleation from existing sites and constant crystal growth. This approach has been widely applied to materials such as PEEK, PA6, PA12 and its composites [38–41]. The isothermal behaviour typically described using the Avrami equation is given by (Eq. 16):

16 $$\:{\mathrm{X}}_{\mathrm{v}\mathrm{c}}=\:{\mathrm{X}}_{\mathrm{v}\mathrm{c}}^{{\infty\:}}\:(1-\mathrm{exp}\left(-\mathrm{K}\left(\mathrm{T}\right)\cdot\:{\mathrm{t}}^{\mathrm{n}}\right))$$

Here, K(T) is the temperature-dependent crystallisation rate constant, n is the Avrami exponent, t is time, and T is the absolute temperature. The volume fraction crystallinity is represented by $\:{\mathrm{X}}_{\mathrm{v}\mathrm{c}}$ while $\:{\mathrm{X}}_{\mathrm{v}\mathrm{c}}^{{\infty\:}}$ denotes the equilibrium crystallinity. A single-mechanism type of model was adopted by Cebe and Hong [38]; however, to achieve good agreement with experimental observations, the Avrami exponent was varied with temperature. Specifically, values of (n) in the range of approximately 2.8 to 3.3 were employed for different crystallisation temperatures, reflecting the temperature dependence of the underlying crystallisation kinetics. If Eq. 16 applied directly to a continuously cooling process, which represents the actual thermal conditions encountered in thermoforming, Automated Fiber Placement (AFP), and Automated Tape Placement (ATP), the Avrami model is not strictly applicable. This limitation arises because the crystallization rate constant, K, is temperature-dependent and therefore changes continuously throughout the cooling cycle, whereas the Avrami equation was originally developed for isothermal crystallization conditions. The standard extension the Nakamura model, which introduces an isokinetic assumption, n treated as constant while K(T) varies, and this allows the isothermal Avrami form to be integrated over a non-isothermal cooling path [42, 43]. This isokinetic assumption is itself the model’s main weakness, since nucleation and growth do not always share the same temperature dependence, and the assumption becomes less reliable across wide cooling-rate ranges. The Nakamura-Ziabicki modification relaxes this by letting the rate function’s characteristic cooling rate itself be temperature-dependent, and it has been shown to track polyamide crystallisation across a much wider range of cooling rates than the basic Nakamura form, which directly relevant to fast-cooling processes like thermoforming [44]. Ozawa’s model offers an alternative non-isothermal formulation based on constant cooling rate rather than isokinetic assumptions, and a recent comparison of Avrami, Ozawa, Mo and Nakamura formulations against fibre-reinforced thermoplastic crystallisation data found no single model performs best across all cooling rates, which reinforces that model choice should be matched to the expected process cooling regime rather than defaulting to isothermal Avrami [45].

#### Thermo-chemical shrinkage model

Shrinkage is an important phenomenon, whether Crystallisation-induced shrinkage in thermoplastic composites or cure-induced shrinkage in thermosets, shrinkage strains along the principal material directions are generally evaluated using micromechanical approaches. A key distinction lies in the material constitutive behaviour: for thermoplastic models, they depend on both temperature and degree of crystallinity, whereas thermosetting systems are governed by temperature and degree of cure. The overall thermal expansion/contraction and shrinkage response of the composites can be predicted through micromechanics-based formulations. For thermosetting resins, chemical shrinkage is typically defined in terms of total volumetric shrinkage V_sh_. By assuming uniform volumetric contraction within a representative unit cell, the resulting isotropic incremental shrinkage strain $\:{\dot{{\upepsilon\:}}}_{\mathrm{r}}^{\mathrm{c}}$ in the resin can be expressed as [46] (Eq. 17):

17 $$\:{\dot{{\upepsilon\:}}}_{\mathrm{r}}^{\mathrm{c}}=\:\sqrt[3]{1+\varDelta\:{\upalpha\:}.{\mathrm{V}}_{\mathrm{s}\mathrm{h}}}-1$$

where $\:\varDelta\:{\upalpha\:}$ represents the change in the degree of cure, and V_sh_ denotes the total volumetric shrinkage of the thermosetting resin. Shrinkage initiates after gelation and is commonly assumed to vary linearly with the degree of cure [47]. The linear relationship between chemical shrinkage and degree of cure assumes shrinkage accrues uniformly across the reaction; this does not hold for resins with strongly nonlinear post-gel shrinkage, where most of the volumetric change happens in a narrow conversion window near gelation [46].

In thermoplastic systems, resin shrinkage can be directly related to the evolution of crystallinity, provided that the densities of the amorphous and crystalline phases are known. The isotropic crystallisation shrinkage strain $\:{\dot{{\upepsilon\:}}}_{\mathrm{r}}^{\mathrm{c}}$ can be derived from the corresponding incremental volumetric shrinkage $\:\varDelta\:{\mathrm{V}}_{\mathrm{c}}$ associated with phase transformation within a representative resin volume element [9]. This formulation links microstructural changes during crystallisation to the resulting macroscopic strain response and can be given as (Eq. 18):

18 $$\:{\dot{{\upepsilon\:}}}_{\mathrm{r}}^{\mathrm{c}}=\:\frac{-1+\:\sqrt{1+\left(\frac{4}{3}\right)\varDelta\:{\mathrm{V}}_{\mathrm{c}}}}{2}$$

### Constitutive material models

This section presents constitutive modelling approaches commonly reported in the literature, namely linear elastic, viscoelastic, and path‑dependent models. The corresponding stress–strain relations and matrix elastic properties are introduced. The effective mechanical properties, along with thermal and chemical shrinkage strains of the composite, are determined using the micromechanics modelling approach.

#### Linear elastic model

In thermoset resin, mechanical stiffness is strongly influenced by the degree of cure. A commonly adopted formulation defines the cure-dependent instantaneous isotropic modulus by partitioning the curing process into three regimes [46]. In the initial stage, the resin is fully uncured and behaves as a viscous fluid, thus exhibiting a constant, very low modulus. In the intermediate regime, the modulus evolves with the degree of cure ($\:{\upalpha\:}$), reflecting progressive network formation and a concurrent reduction in specific volume due to chemical shrinkage. In the final stage, corresponding to a fully cured state, the modulus reaches a constant maximum value, with no further chemical shrinkage occurring [33, 46]. The modulus generation as function of temperature can be expressed as (Eq. 19):

19 $$\:{\mathrm{E}}_{\mathrm{r}}=\left(1-{\upalpha\:}\right){\mathrm{E}}_{\mathrm{r}}^{0}+\:{\upalpha\:}{\mathrm{E}}_{\mathrm{r}}^{{\infty\:}}+\:{\upgamma\:}{\upalpha\:}(1-{\upalpha\:})({\mathrm{E}}_{\mathrm{r}}^{{\infty\:}}-\:{\mathrm{E}}_{\mathrm{r}}^{0})$$

Here, $\:{\mathrm{E}}_{\mathrm{r}}^{0}$ and $\:{\mathrm{E}}_{\mathrm{r}}^{{\infty\:}}$ are represented as uncured and fully cured resin modulus, respectively. In general, for simplicity, uncured resin modulus taken as the 1000 times less than the cured resin modulus [34, 48]. To account for thermal effects, this relationship has been extended within the CHILE approach, incorporating temperature dependence to capture both cure-induced stiffening and thermal softening [33, 49]. This modification introduces critical temperatures associated with the onset and completion of the glass transition and defines the modulus as a function of the difference between the instantaneous glass transition temperature and the current resin temperature. The temperature-dependent modulus generation equation can be expressed as (Eq. 20):

20 $$\:\begin{array}{c}{\:\mathrm{E}}_{\mathrm{r}}=\:\left\{\begin{array}{cc}{\mathrm{E}}_{\mathrm{r}}^{0}&\:{\mathrm{T}}^{\mathrm{*}}<{\mathrm{T}}_{\mathrm{c}1}\\\:\left(\frac{{\mathrm{T}}_{\mathrm{c}2}-{\mathrm{T}}^{\mathrm{*}}}{{\mathrm{T}}_{\mathrm{c}2}-{\mathrm{T}}_{\mathrm{c}1}}\right){\mathrm{E}}_{\mathrm{r}}^{0}+\left(\frac{{\mathrm{T}}^{\mathrm{*}}-{\mathrm{T}}_{\mathrm{c}1}}{{\mathrm{T}}_{\mathrm{c}2}-{\mathrm{T}}_{\mathrm{c}1}}\right){\mathrm{E}}_{\mathrm{r}}^{{\infty\:}}&\:{\mathrm{T}}_{\mathrm{c}1}\le\:{\mathrm{T}}^{\mathrm{*}}<{\mathrm{T}}_{\mathrm{c}2}\\\:{\mathrm{E}}_{\mathrm{r}}^{{\infty\:}}&{\mathrm{T}}^{\mathrm{*}}\ge\:{\mathrm{T}}_{\mathrm{c}2}\end{array}\right.\\\:\mathrm{w}\mathrm{h}\mathrm{e}\mathrm{r}\mathrm{e}\:{\mathrm{T}}^{\mathrm{*}}={\mathrm{T}}_{\mathrm{g}}-\mathrm{T}\end{array}$$

**Fig. 2:**
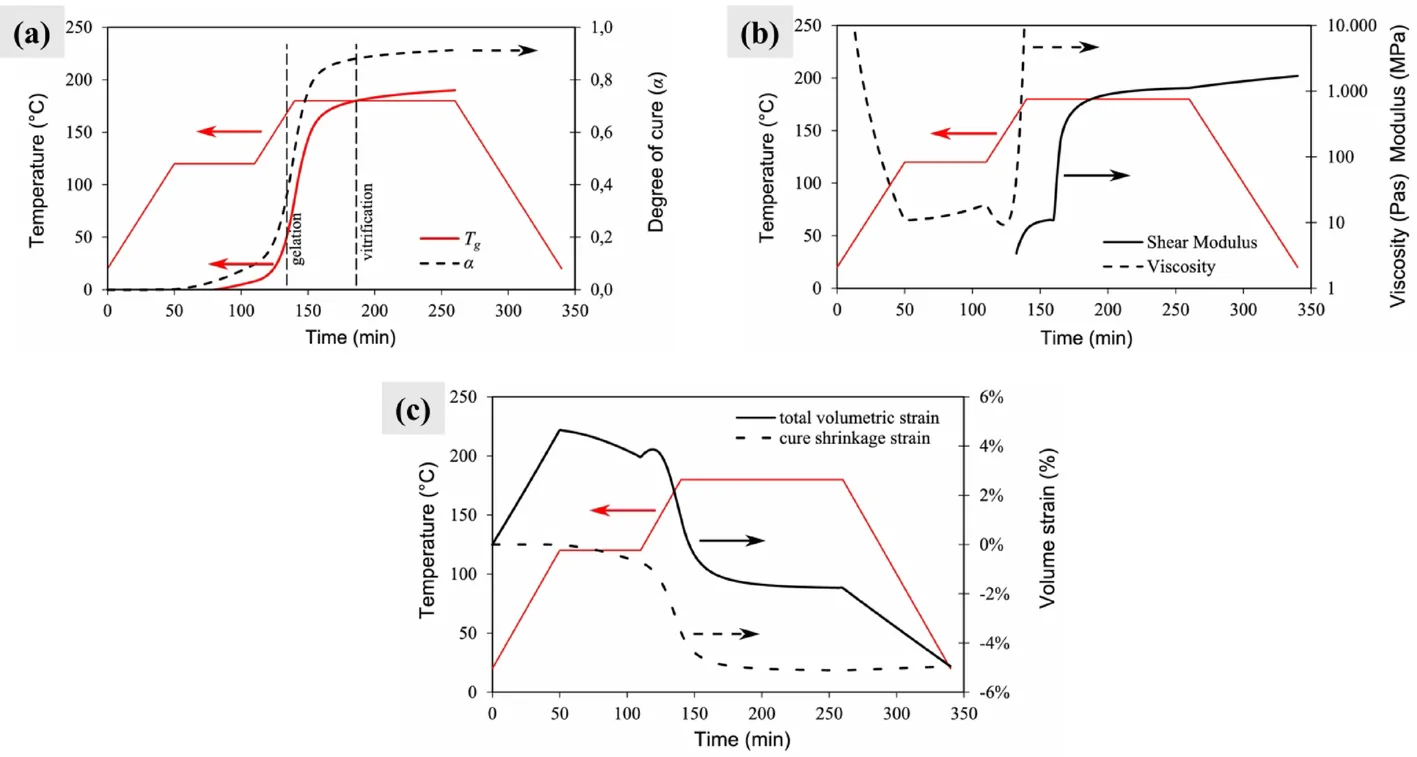
Evolution of epoxy (HEXEL 8552) resin properties: **a** degree of cure and glass transition temperature [50–53], **b** viscosity and modulus [52], and **c** volumetric strain [52]

Here, T_c1_ and T_c2_ are defined as the window over which modulus is assumed to increase linearly with degree of cure; the assumption of instantaneous, temperature-independent property development inside that window is reasonable away from the glass transition, but becomes progressively less accurate as the cure temperature gets close to T_c2_, where actual modulus development is rate-dependent rather than instantaneous [33, 49]. In other words, these are critical temperatures at which the modulus starts and stops growing, respectively. The evolution of key material properties for a typical epoxy system, such as Hexcel 8552 [50], illustrates these mechanisms as shown in Fig. 2. Figure 2a represents the applied temperature history (Tg) and the evolution of the degree of cure over time, indicating the increase in cure during the heating and holding stages and the corresponding evolution of the glass-transition temperature. As curing progresses in Fig. 2b, the resin viscosity and the shear modulus increase significantly as the material develops mechanical stiffness, which can be reflected in Eq. 20. The cure shrinkage develops mainly during the curing stage and remains after the material has reached a highly cured state, as represented in Fig. 2c. The whole process demonstrates the basis of the CHILE approaches that treats these three stages independently, rather than through a shared degree-of-cure state variable.”

Unlike thermosetting systems, where stiffness development is primarily governed by the progression of cure and glass-transition evolution, thermoplastic composites derive their evolving mechanical response from temperature-dependent crystallisation and solidification phenomena occurring during cooling. In thermoplastics, residual stress evaluation requires ply properties that vary with time, temperature, and crystallinity, and depend on constituent behaviour and volume fractions. For semi-crystalline composites (e.g., APC-2), the matrix response is governed by amorphous and crystalline phases and their evolving crystallinity. A standard linear solid (SLS) kinetic–viscoelastic model given by Lawrence, Seferis [9] represents this behaviour using storage and loss flexural compliances ($\:{\mathrm{S}}^{{\prime\:}}$ and $\:{\mathrm{S}}^{{\prime\:}{\prime\:}}$ respectively) as functions of crystallinity (X_vc_) and can be expressed as (Eqs. 21, 22, 23, 24, 25):

21 $$\:{\mathrm{S}}^{{\prime\:}}=\:{\mathrm{S}}_{\mathrm{a}\mathrm{m}}^{{\prime\:}}\left(1-{\mathrm{X}}_{\mathrm{v}\mathrm{c}}\right)+{\mathrm{S}}_{\mathrm{c}\mathrm{r}}^{{\prime\:}}\left({\mathrm{X}}_{\mathrm{v}\mathrm{c}}\right)$$

22 $$\:{\mathrm{S}}^{{\prime\:}{\prime\:}}={\mathrm{S}}_{\mathrm{a}\mathrm{m}}^{{\prime\:}{\prime\:}}\left(1-{\mathrm{X}}_{\mathrm{v}\mathrm{c}}\right)+{\mathrm{S}}_{\mathrm{c}\mathrm{r}}^{{\prime\:}{\prime\:}}\left({\mathrm{X}}_{\mathrm{v}\mathrm{c}}\right)$$

where,

23 $$\:{\mathrm{S}}_{\mathrm{a}\mathrm{m}}^{{\prime\:}}=\:{\mathrm{S}}_{\mathrm{u}\mathrm{a}}+({\mathrm{S}}_{\mathrm{r}\mathrm{a}}-{\mathrm{S}}_{\mathrm{u}\mathrm{a}}){\left(\mathrm{cos}{\upvarphi\:}\right)}^{\mathrm{a}}\mathrm{cos}\left({\upvarphi\:}\mathrm{a}\right)$$

24 $$\:{\mathrm{S}}_{\mathrm{a}\mathrm{m}}^{{\prime\:}{\prime\:}}=\:({\mathrm{S}}_{\mathrm{r}\mathrm{a}}-{\mathrm{S}}_{\mathrm{u}\mathrm{a}}){\left(\mathrm{cos}{\upvarphi\:}\right)}^{\mathrm{a}}\mathrm{sin}\left({\upvarphi\:}\mathrm{a}\right)$$

25 $$\:{\upvarphi\:}=\mathrm{a}\mathrm{r}\mathrm{c}\mathrm{tan}\left({\upomega\:}{{\uptau\:}}_{\mathrm{a}\mathrm{m}}\right)$$

Here, $\:{\mathrm{S}}_{\mathrm{a}\mathrm{m}}^{{\prime\:}}$, ${\mathrm{S}}_{{{\mathrm{am}}}}^{{\prime \prime }}$, $\:{\mathrm{S}}_{\mathrm{c}\mathrm{r}}^{{\prime\:}}$, ${\mathrm{S}}_{{{\mathrm{cr}}}}^{{\prime \prime }}$ are the amorphous and crystalline storage and loss flexural compliances, respectively. $\:{\mathrm{S}}_{\mathrm{u}\mathrm{a}}$, $\:{\mathrm{S}}_{\mathrm{r}\mathrm{a}}$ are the unrelaxed and relaxed amorphous compliances. The parameter a (varies 0 to 1) accounts for the shift in the peak of the dynamic loss modulus with crystallinity, $\:{\upomega\:}$ is the angular frequency and $\:{{\uptau\:}}_{\mathrm{a}\mathrm{m}}$ is the amorphous retardation time. The crystalline phase is assumed to have constant storage compliance and negligible loss compliance, as it does not exhibit relaxation. The temperature dependence of the retardation time was described using two models: the Arrhenius (Andrade) relation (Eq. 26) below the glass transition temperature and the WLF equation (Eq. 27) above the glass transition temperature and shown as:

26 $$\:{\uptau\:}=\:{{\uptau\:}}_{0}\:\mathrm{e}\mathrm{x}\mathrm{p}\left[\frac{{\mathrm{E}}_{\mathrm{a}}}{\mathrm{R}}\:\left(\frac{1}{\mathrm{T}}-\frac{1}{{\mathrm{T}}_{0}}\right)\right]$$

27 $$\:\mathrm{log}\left(\frac{{{\uptau\:}}_{\mathrm{a}\mathrm{m}}}{{{\uptau\:}}_{0}}\right)=\frac{{-\mathrm{C}}_{1}(\mathrm{T}-{\mathrm{T}}_{0})}{{\mathrm{C}}_{2}+(\mathrm{T}-{\mathrm{T}}_{0})}$$

where the reference temperature T_0_ corresponds to the reference retardation time $\:{{\uptau\:}}_{0}$, with E_a_ and R denoting the activation energy and the universal gas constant, respectively. C_1_ and C_2_ were determined empirically from time–temperature superposition experiments. For semicrystalline polymers, accurate characterisation above the glass transition is challenging due to recrystallisation [9]. The dynamic storage and loss compliances are related to the matrix modulus (E_r_) through the storage modulus (E’) as shown in Eq. 28. Assuming a constant matrix Poisson’s ratio (v_r_), the time-dependent matrix modulus is linked to the instantaneous shear modulus (G_r_) through the isotropic elasticity relation and can be represented as (Eqs. 28, 29):

28 $$\:{\mathrm{E}}_{\mathrm{r}}\approx\:\:{\mathrm{E}}^{{\prime\:}}=\:\frac{\mathrm{S}}{{{\mathrm{s}}^{{\prime\:}}}^{2}+{{\mathrm{s}}^{{\prime\:}{\prime\:}}}^{2}}\:$$

29 $$\:{\mathrm{G}}_{\mathrm{r}}=\:\frac{{\mathrm{E}}_{\mathrm{r}}}{2(1+{\mathrm{v}}_{\mathrm{r}})}$$

**Fig. 3:**
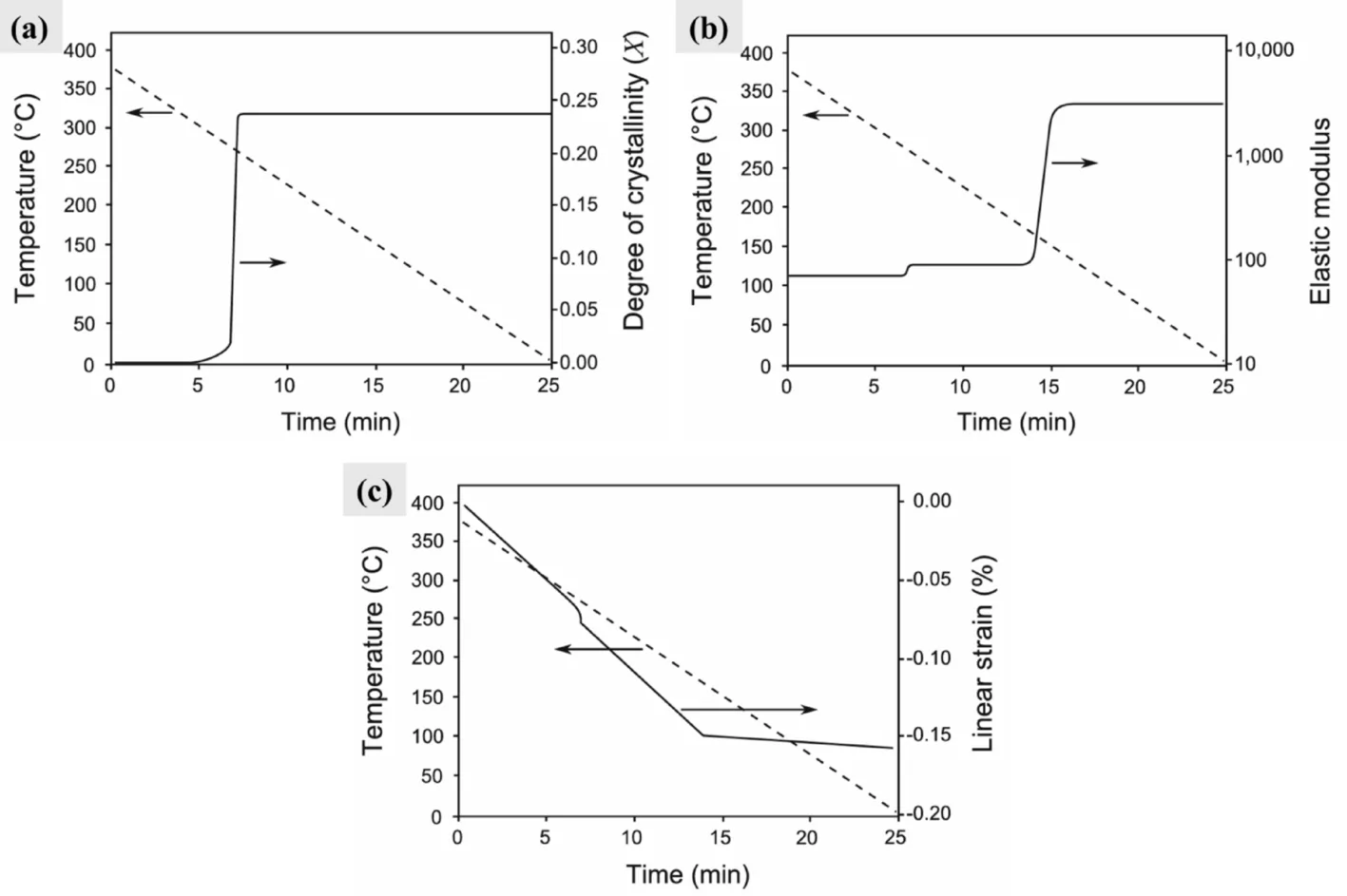
Development of a PEEK thermoplastic resin properties: **a** degree of crystallinity, **b** elastic modulus, and **c** linear strain [10]

The SLS model predicts the resin modulus as a function of temperature and crystallinity, analogous to the CHILE model for thermosets. Figure 3 illustrates the evolution of crystallinity, modulus, and linear shrinkage of PEEK during cooling. Crystallinity peaks ~ 7 min after cooling from 375 °C (2 °C/min), resulting in minor changes in modulus and shrinkage. A sharp increase in modulus (≈ two orders of magnitude) occurs at the glass transition (~ 15 min), marking the transition from rubbery to glassy behaviour, accompanied by a change in coefficient of thermal expansion reflected in the shrinkage slope.

In the micromechanical analysis, the macro stress generated during processing was calculated by following a model given by Heinrich, Aldridge [54] (Eq. 30):

30 $$\:{\upsigma\:}\left(\mathrm{t},\mathrm{s}\right)=\mathrm{E}\left(\mathrm{s}\right) \cdot {{\upepsilon\:}}_{\mathrm{t}\mathrm{o}\mathrm{t}\mathrm{a}\mathrm{l}}(\mathrm{t},\mathrm{s})$$

where $\:{\upsigma\:}\left(\mathrm{t},\mathrm{s}\right)$ is stress at time t in the network created at time s, $\:{{\upepsilon\:}}_{\mathrm{t}\mathrm{o}\mathrm{t}\mathrm{a}\mathrm{l}}(\mathrm{t},\mathrm{s})$ is the sum of all incremental strains at time t in the network formed at time s, and E(s) is the elastic modulus in the network formed at time s. The sum of mechanical$\:\:\left({{\upepsilon\:}}^{\mathrm{m}\mathrm{e}\mathrm{c}\mathrm{h}}\right)$, thermal and shrinkage strains ($\:{{\upepsilon\:}}_{\mathrm{c}})$ gives the total strain [33, 49], i.e. (Eq. 31).

31 $$\:{{\upepsilon\:}}_{\mathrm{t}\mathrm{o}\mathrm{t}\mathrm{a}\mathrm{l}}\left(\mathrm{t},\mathrm{s}\right)=\:{{\upepsilon\:}}^{\mathrm{m}\mathrm{e}\mathrm{c}\mathrm{h}}\left(\mathrm{t},\mathrm{s}\right)+\:{\upalpha\:}\left(\mathrm{s}\right) \cdot \varDelta\:\left({\uptheta\:}\left(\mathrm{t}\right)-{\uptheta\:}\left(\mathrm{s}\right)\right)+\:{{\upepsilon\:}}_{\mathrm{c}}\left(\mathrm{s}\right)$$

where θ represents the temperature in the formed network. $\:{\upalpha\:}\left(\mathrm{s}\right)\:$and $\:{{\upepsilon\:}}_{\mathrm{c}}\left(\mathrm{s}\right)\:$is the coefficient of thermal expansion and shrinkage strain of the network created at time s, respectively.

#### Viscoelastic model

Viscoelastic constitutive models are widely employed to predict residual stresses and deformations by accounting for stress relaxation during polymerisation. Such behaviour is particularly significant in composite processing at high temperatures and extended curing times, as in RTM [34]. These models are commonly formulated in integral form [55]. Assuming zero prior strain history at t = 0, the linear viscoelastic response can be expressed as a function of temperature and the evolving degree of cure or crystallisation ($\:{\upalpha\:}$) and given by [55–60] (Eq. 32):

32 $$\:{\boldsymbol{\upsigma}}_{\mathrm{i}}\left(\mathrm{t}\right)={\int}_{0}^{\mathrm{t}}{\mathbf{C}}_{\mathrm{i}\mathrm{j}}({\upalpha},\mathrm{T},{{\upxi}}^{\mathrm{t}}-{{\upxi}}^{{\prime}})\frac{\partial\:{\boldsymbol{\upepsilon}}_{\mathrm{j}}^{\mathrm{e}\mathrm{f}\mathrm{f}}}{\partial\:{{\uptau}}_{i}}\mathrm{d}{\uptau}$$

Where, $\:{\boldsymbol{\upsigma\:}}_{\mathrm{i}}$ and $\:{{\upepsilon\:}}_{\mathrm{j}}$ represents the stress and strain tensor, respectively. $\:{{\upxi}}^{\mathrm{t}}$ and $\:{{\upxi}}^{{\prime}}$ are the current and past reduced time, respectively and can be expressed as (Eq. 33, Eq. 34):

33 $$\:{{\upxi}}^{\mathrm{t}}={\int}_{0}^{\mathrm{t}}\frac{\mathrm{d}\mathrm{s}}{{\mathrm{a}}_{\mathrm{T}}({{\upalpha}},\mathrm{T})}$$

34 $$\:{{\upxi}}^{{\prime}}={\int}_{0}^{{\uptau}}\frac{\mathrm{d}\mathrm{s}}{{\mathrm{a}}_{\mathrm{T}}{{\upalpha}},\mathrm{T})}$$

**Fig. 4:**
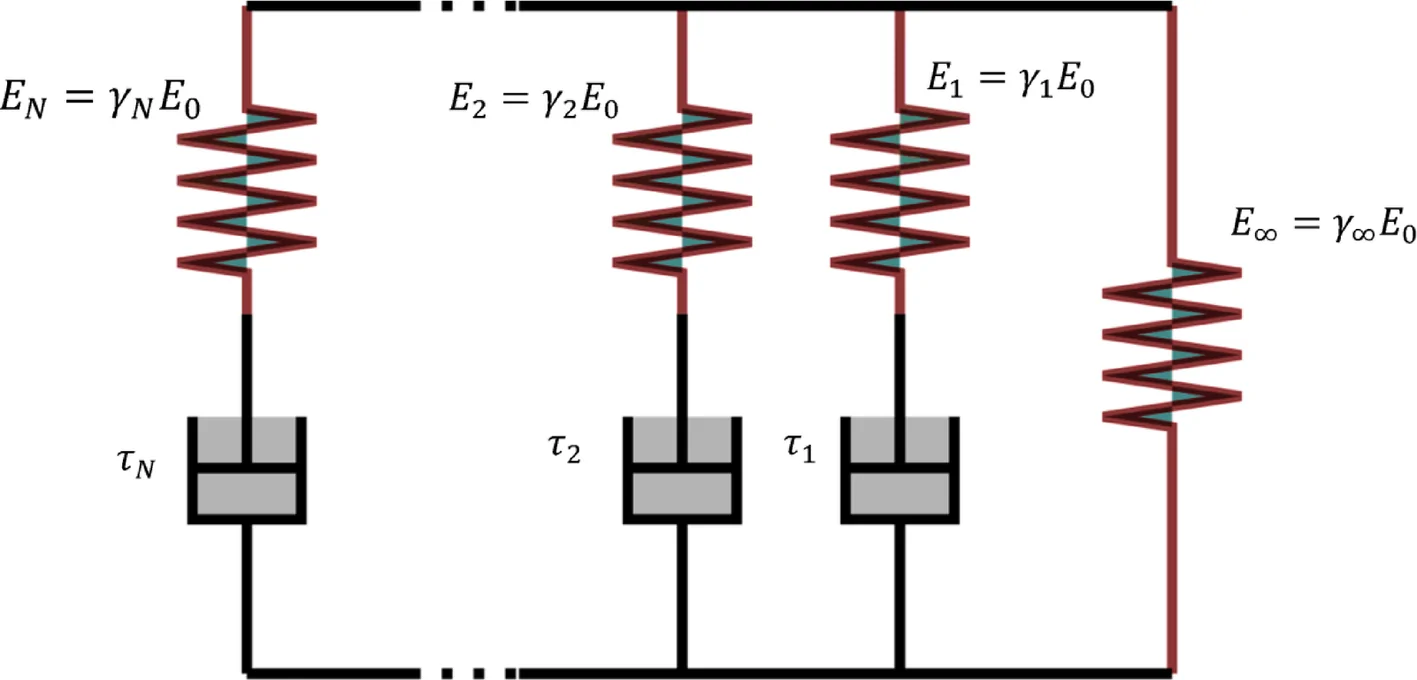
Configuration of the generalised Maxwell model [61]

Where, $\:{\mathrm{a}}_{\mathrm{T}}$ is the shift factor. The relaxed stiffness tensor (C_ij_) and relaxed modulus (E_r_) of the matrix can be obtained from the generalised Maxwell equations, comprising Maxwell elements (spring–dashpot units) in parallel with an equilibrium spring representing the fully relaxed state, as shown in Fig. 4. Accordingly, it is approximated using a Prony series expansion [56] and can be represented as (Eq. 35, Eq. 36):

35 $${\mathrm{C}}_{{{\mathrm{ij}}}} \left( {\alpha ,\xi } \right) = \:{\mathrm{C}}_{{{\mathrm{ij}}}}^{0} + \left( {{\mathrm{C}}_{{{\mathrm{ij}}}}^{1} - {\mathrm{C}}_{{{\mathrm{ij}}}}^{0} } \right)\sum\limits_{{{\mathrm{im}} = 1}}^{{\mathrm{n}}} {{\mathrm{W}}_{{\mathrm{m}}} } {\mathrm{exp}}\left( { - \frac{{\xi (\alpha ,{\mathrm{T}})}}{{\tau _{{{\mathrm{im}}}} \left( {{\upalpha}} \right)}}} \right) $$

36 $${\mathrm{E}}_{{\mathrm{r}}} \left( {\alpha ,{\mathrm{T}},{\mathrm{t}}} \right) = \:{\mathrm{E}}_{{\mathrm{r}}}^{0} + \left( {{\mathrm{E}}_{{\mathrm{r}}}^{0} - {\mathrm{E}}_{{\mathrm{r}}}^{1} } \right)\:\sum\limits_{{\mathrm{i}}}^{{\mathrm{n}}} {{\mathrm{W}}_{{{\mathrm{mi}}}} } {\mathrm{exp}}\left( { - \frac{{\xi (\alpha ,{\mathrm{T}})}}{{\tau _{{{\mathrm{im}}}} \left( {{\upalpha}} \right)}}} \right)$$

Where, $\:{\mathrm{C}}_{\mathrm{i}\mathrm{j}}^{0}$ and $\:{\mathrm{C}}_{\mathrm{i}\mathrm{j}}^{1}$ are the fully relaxed and unrelaxed stiffness, respectively. Fully relaxed and unrelaxed modulus are represented as $\:{\mathrm{E}}_{\mathrm{r}}^{0}$ and $\:{\mathrm{E}}_{\mathrm{r}}^{1}$ respectively. The Prony series shift factor $\:{\mathrm{a}}_{\mathrm{T}}$ and the individual relaxation times τ_i_ are only physically meaningful if enough terms are included to span the material’s actual relaxation spectrum; truncating the series under-represents long-term relaxation and gives an artificially stiff stress prediction at long hold times [55]. The parallel spring-dashpot arrangement in Fig. 4 illustrates why the Prony series (Eqs. 35–36) is generally more computationally demanding than CHILE as each additional Maxwell element adds its own relaxation time and weighting factor, so capturing a broad relaxation spectrum trades directly against the number of internal state variables.

The generalised Maxwell/Prony series representation in Eqs. 32–36 is a linear viscoelastic formulation, and its accuracy in predicting long-term relaxation is bounded by how completely the fitted relaxation times span the material’s actual relaxation spectrum; too few Prony terms under-predict long-term stress relaxation and give an artificially high residual stress at long hold times [56]. More fundamentally, the linear formulation cannot capture strain-rate- or amplitude-dependent nonlinearity, which becomes relevant near the glass transition or under the large strains that can arise during tool removal or post-cure handling. A recent comparison across several viscoelastic solids found a nonlinear time-dependent-viscosity formulation to outperform generalised Maxwell/Prony specifically when predicting outside the strain and time range the model was calibrated on, using far fewer fitted parameters, which is exactly the extrapolation regime process simulation often needs, since a full experimental relaxation dataset spanning the whole cure-to-service timescale is rarely available [62].

#### Path dependent model

A simplified viscoelastic formulation replaces rate dependence with path dependence for describing material behaviour. In this approach, the resin properties transition abruptly from a rubbery to a glassy state at the glass transition temperature (T_g_), leading to a corresponding step change in composite properties. The glass transition is explicitly incorporated into the stiffness matrix and stress–strain relations, forming the basis of the constitutive model [48, 63–65]. The corresponding stress-strain equations can be represented as (Eq. 37):

37 $$\:{{\upsigma\:}}_{\mathrm{i}}=\:\left\{\begin{array}{c}\begin{array}{cc}{\mathrm{C}}_{\mathrm{i}\mathrm{j}}^{\mathrm{r}}{{\upepsilon\:}}_{\mathrm{j}}&\:{\mathrm{T}}^{\mathrm{*}}\:\ge\:{\mathrm{T}}_{\mathrm{g}}\left({\upalpha\:}\right)\end{array}\\\:\begin{array}{cc}{\mathrm{C}}_{\mathrm{i}\mathrm{j}}^{\mathrm{g}}{{\upepsilon\:}}_{\mathrm{j}}-{\left[({\mathrm{C}}_{\mathrm{i}\mathrm{j}}^{\mathrm{g}}-{\mathrm{C}}_{\mathrm{i}\mathrm{j}}^{\mathrm{r}}){{\upepsilon\:}}_{\mathrm{j}}\right]}_{\mathrm{t}=\:{\mathrm{t}}_{\mathrm{v}\mathrm{i}\mathrm{t}}}&\:{\mathrm{T}}^{\mathrm{*}}<{\mathrm{T}}_{\mathrm{g}}\left({\upalpha\:}\right)\end{array}\end{array}\right.$$

where $\:{\mathrm{t}}_{\mathrm{v}\mathrm{i}\mathrm{t}}$ denotes the time of the most recent rubber–glass transition (vitrification), and the subscripts $\:\mathrm{r}$ and $\:\mathrm{g}$ represent the rubbery and glassy states, respectively.

A common assumption in process simulation is that constitutive models developed for one polymer system can be transferred directly to another following limited parameter adjustment. In practice, however, transferability is generally restricted to the mathematical structure of the model rather than the calibrated parameter values themselves. For thermosetting polymers, cure-kinetics parameters, activation energies, reaction orders, heat of reaction, glass-transition evolution and viscosity-growth parameters are strongly dependent on resin chemistry and therefore typically require complete recalibration using DSC, DMA and rheological measurements. Similarly, in thermoplastic systems, crystallisation-kinetics parameters, Avrami or Nakamura coefficients, melting and crystallisation temperatures, relaxation spectra and crystallinity-dependent mechanical properties are material-specific and often processing-route dependent. As a result, constitutive parameters calibrated for one thermoplastic system cannot generally be transferred directly to another without additional material characterisation and validation. In contrast, the general constitutive framework, such as CHILE, viscoelastic Maxwell-Prony formulations, path-dependent models or crystallisation-kinetics-based approaches, can usually be transferred between material systems because they represent common physical mechanisms rather than specific material behaviour. Consequently, model transferability is best viewed as transfer of methodology rather than transfer of calibrated material parameters, and reliable predictions generally require material-specific characterisation before implementation in process simulations.

The constitutive models reviewed in this section reflect a consistent trade-off between physical completeness and computational cost. Simplified formulations such as the base cure-dependent modulus model [46], CHILE [33,49], and path-dependent models [8,63 –65] remain the more commonly used approaches, while the full linear viscoelastic Prony treatment [55–60] becomes particularly relevant for processes involving extended curing times at elevated temperature, such as RTM [59]. What the literature does not conclusively establish is exactly how much accuracy is lost by the simpler models outside these extended-cure scenarios, a question the studies reviewed here do not directly settle through head-to-head comparison. The constitutive models described in Sect. 2.1 and 2.2 provide the fundamental relationships required to simulate thermal, chemical and mechanical behaviour during composite processing. Although these models differ in their underlying assumptions, required material characterisation and computational requirements, they form the basis of most process-simulation frameworks reported in the literature. For clarity, Table 1 summarises the main constitutive models reviewed in Sect. 2.1 and 2.2, together with their governing equations, key assumptions, required material inputs and relative computational cost.

**Table 1:** Review of constitutive models used in the composite process modelling

Model	Equation(s)	Key assumptions	Required material/data inputs	Computational cost
Thermo-kinetics/Fourier heat transfer [23–25]	Eq. 1	Heat generation from cure/crystallisation reaction can be captured as an internal source term in the transient heat equation	Thermal conductivity, specific heat, density, reaction exotherm/crystallisation data (e.g., DSC)	Low
Kamal-Sourour autocatalytic cure kinetics [3–5, 23]	Eqs. 9–11	Cure rate follows a single autocatalytic reaction mechanism	Isothermal/dynamic DSC data; kinetic parameters	Low
Avrami crystallisation kinetics [6–8]	Eq. 16	Isothermal, isokinetic nucleation and spherulitic growth	Isothermal DSC, XRD	Low
SLS kinetic-viscoelastic model [9]	Eqs. 21–29	Resin modulus develops as a function of both temperature and crystallinity, analogous to CHILE for thermosets	DMA storage/loss compliance data, time-temperature superposition (C1, C2). crystallinity data	Medium
Cure-dependent modulus (base model) [46]	Eq. 19	Modulus develops through three regimes (uncured/curing/fully cured) as a function of degree of cure alone, no temperature dependence	DSC, modulus measurements, cure data	Low
CHILE (temperature-extended) [33, 49]	Eq. 20	Extends the base model with temperature dependence via critical temperatures Tc1/Tc2; instantaneous property change assumed	DSC, limited DMA. temperature-dependent material properties	Low
Generalized Maxwell/Prony series viscoelastic model [55–60] (Fig. 4 model diagram: [61])	Eqs. 32–36	Linear viscoelasticity, relaxation representable as a sum of exponential (Maxwell) terms	Multi-frequency, multi-temperature DMA to populate the full relaxation spectrum	Medium-High
Path-dependent model [48, 63–65]	Eq. 37	Rate dependence replaced by a step change in properties at vitrification (Tg) rather than continuous relaxation	Tg as a function of degree of cure, rubbery/glassy stiffness	Low

Table 1 highlights the diversity of constitutive approaches used in composite process modelling, ranging from relatively simple thermal and kinetic formulations to more advanced viscoelastic frameworks. While low-order models remain attractive because of their efficiency and limited input requirements, higher-fidelity approaches generally offer improved representation of evolving material behaviour during processing. The constitutive descriptions summarised in Table 1 form the basis for evaluating the mechanisms responsible for residual stress generation and dimensional distortion in composite structures, which are discussed in the Sect.  2.3.

### Residual stress and deformation

Process-induced residual stresses and deformations are inherent to composite manufacturing and have been widely studied. Prior work primarily focuses on either elucidating the underlying physical mechanisms or predicting the resulting distortions using analytical and numerical models. A comprehensive understanding of these stresses is essential, as they directly control residual shape distortions and dimensional tolerance. Residual stresses originate from interacting mechanisms such as thermal anisotropy, resin shrinkage, tool–part interaction, consolidation effects, fibre volume fraction gradients, moisture swelling, prepreg variability, and gradients in temperature and degree of cure or crystallisation.

**Fig. 5:**
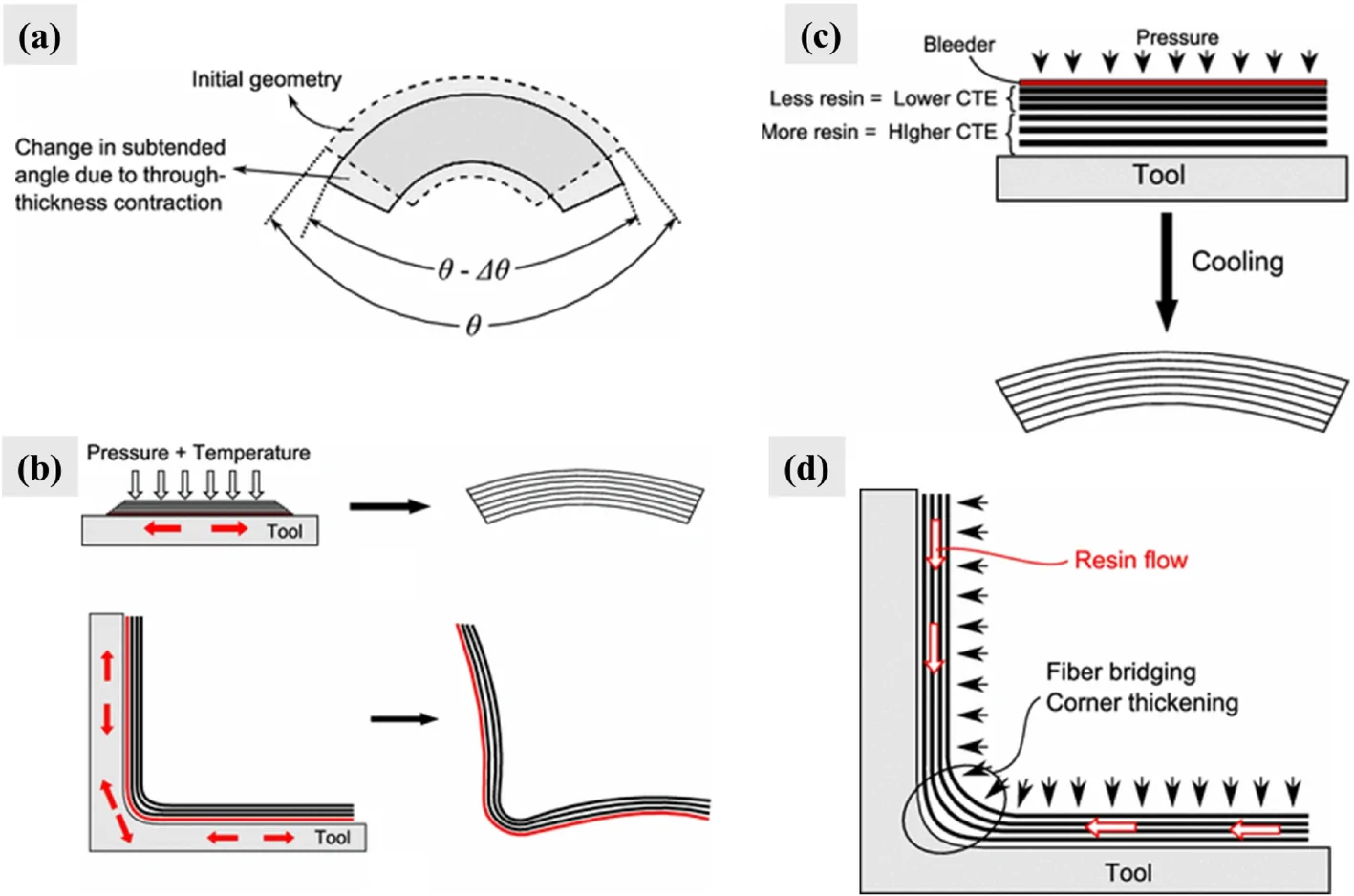
**a** spring in deformation pattern due to a change in enclosed angle θ, **b** effect of tool part interaction in flat and corner sections, **c** warpage due to resin flow in flat laminates, **d** corner thickening due to resin flow [10]

The mismatch in the coefficient of thermal expansion (CTE) between fibres and matrix is a primary source of process-induced residual stresses in fibre-reinforced composites, acting across both micro- and macro-scales. Typically, fibres exhibit significantly lower CTE than the surrounding resin, leading to pronounced thermal anisotropy at the laminate level. As a result, composite parts tend to expand or contract more in matrix-dominated directions than along fibre-dominated orientations [19]. A balanced and symmetric flat laminates may not exhibit out-of-plane distortion in the absence of tooling constraints, while curved geometries (such as L-shape or c-shape) are particularly susceptible to distortion due to differential thermal contraction between the through-thickness and in-plane directions [10]. This behaviour manifests as a reduction in the enclosed angle, commonly referred to as spring-in (as shown in Fig. 5a). The magnitude of spring-in ($\:\varDelta\:{\uptheta\:})\:$has been analytically described in early models proposed by Nelson and Cairns [66], which expresses it as a function of the subtended angle θ, circumferential and radial CTEs ($\:{{\upalpha\:}}_{{\uptheta\:}}\:\mathrm{a}\mathrm{n}\mathrm{d}\:{{\upalpha\:}}_{\mathrm{R}})$, and temperature change $\:(\varDelta\:\mathrm{T})$ and given in Eq. 38:

38 $$\:\varDelta\:{\uptheta\:}=\:{\uptheta\:}\frac{({{\upalpha\:}}_{{\uptheta\:}}-{{\upalpha\:}}_{\mathrm{R}})\varDelta\:\mathrm{T}}{1+{{\upalpha\:}}_{\mathrm{R}}\varDelta\:\mathrm{T}}$$

In thermoplastic composites, volumetric shrinkage is primarily governed by crystallisation during cooling, whereas in thermosetting systems, shrinkage predominantly occurs during the curing stage prior to cool-down. In thermosets, the polymerisation process transforms the initially liquid resin into a rigid, cross-linked network through chemical reactions, increasing material density and reducing volume [67]. This cure-induced shrinkage is confined to the polymerisation phase and effectively ceases once the reaction reaches completion. Owing to the constraining effect of reinforcing fibres, the resulting shrinkage is anisotropic, with significantly higher strains developing in the transverse and through-thickness directions compared to the fibre-aligned direction [10]. Consequently, the influence of chemical shrinkage on residual stress development and dimensional distortion is analogous to that of thermally induced contraction, and it can be incorporated within similar analytical frameworks. To account for this contribution in the prediction of spring-in behaviour, modified formulations have been proposed by Radford and Diefendorf [68] in which an additional term representing cure shrinkage is introduced into the governing expression (i.e. Equation 38), enabling a more comprehensive description of angular distortion. The modified expression accounts for the cure shrinkage given by (Eq. 39):

39 $$\:\varDelta\:{\uptheta\:}=\:{\uptheta\:}\left[\frac{({{\upalpha\:}}_{{\uptheta\:}}-{{\upalpha\:}}_{\mathrm{R}})\varDelta\:\mathrm{T}}{1+{{\upalpha\:}}_{\mathrm{R}}\varDelta\:\mathrm{T}}+\:\frac{{{\upepsilon\:}}_{{\uptheta\:}}-\:{{\upepsilon\:}}_{\mathrm{R}}}{1+\:{{\upepsilon\:}}_{\mathrm{R}}}\right]$$

Here, $\:{{\upepsilon\:}}_{{\uptheta\:}}$ and $\:{{\upepsilon\:}}_{\mathrm{R}}$ representing the in-plane shrinkage and through-thickness chemical strain, respectively.

For thin and moderately curved composite components, residual stress development is predominantly governed by thermoelastic strain accumulation resulting from differences in the coefficients of thermal expansion of the fibre, matrix, and tooling materials. Unlike other stress-generating mechanisms, these thermal mismatch strains act over the entire post-cure cooling period, making them the principal contributor to the final residual stress state [69]. Chemical shrinkage strain becomes proportionally more significant in thick sections and highly constrained geometries, where through-thickness cure gradients mean that different regions shrink at different times relative to their neighbours, thereby locking in additional stress beyond what CTE mismatch alone would produce [20, 66, 68]. Tool-part interaction, frictional or adhesive constraint at the tool surface, does not generate stress independently but amplifies whichever of the above already present, by stopping the part from relaxing freely during cooldown; its contribution is therefore geometry- and tooling-material-dependent rather than a fixed fraction [10]. Fibre-volume-fraction gradients arising from resin flow during cure act in a similar way, as a secondary amplifier, locally changing the effective CTE and modulus rather than being an independent stress source on its own.

Resin flow represents a key multiphysical phenomenon in composite manufacturing, significantly influencing the evolution of stress–strain fields, microstructural uniformity, and final part geometry. It governs the spatial distribution of fibre volume fraction (FVF), which directly determines local elastic properties required for accurate stress analysis. During processing, resin migration leads to the formation of resin-rich and resin-deficient regions, thereby introducing heterogeneity in mechanical response. Additionally, resin pressure and flow pathways play a critical role in void formation and transport within the laminate [69]. In processes such as vacuum bagging, the use of bleeder materials to enhance fibre volume fraction promotes through-thickness resin drainage, resulting in FVF gradients across the laminate thickness. Consequently, flat laminates often exhibit resin-rich zones near the tool surface and resin-poor regions towards the bag side. Since the effective coefficient of thermal expansion (CTE) depends on the local FVF, these gradients induce asymmetric thermal contraction during cooling, which, in turn, generates residual stresses and macroscopic warpage, as shown in Fig. 5c. Although compaction mechanisms in curved components share similarities with flat laminates, additional complexities arise due to fibre bridging at corners. Frictional resistance between prepreg plies inhibits proper conformance to the tool geometry, reducing effective compaction pressure in these regions and creating low-pressure zones that become infiltrated by resin. This results in localised corner thickening, elevated resin content, and increased through-thickness CTE, thereby amplifying spring-in effects, particularly in components with tight radii, as shown in Fig. 5d. Figure 5d illustrates that corner thickening may arise from a coupled mechanism, as fibre bridging limits local compaction and promotes resin redistribution into the corner region. The resulting local increase in resin content and laminate thickness changes the local thermal and mechanical response and can contribute to spring-in during cooling, as described in Fig. 5a-c. This highlights that a process model validated only against flat-panel spring-in data cannot be assumed to transfer directly to cornered geometries without separately capturing this resin-redistribution effect.

Taken together, the literature reviewed in this section shows reasonable consensus that CTE mismatch is the dominant thermal driver of spring-in for thin, mildly curved geometries [19], but there is less agreement on the relative weighting of chemical shrinkage against tool-part interaction in thick or highly constrained sections, with different studies reporting each as dominant depending on tooling material and part geometry [20, 46, 66]. A genuinely unresolved question is whether a single generalised framework can predict this relative weighting a priori for an arbitrary geometry, or whether case-specific FE calibration will remain necessary; the models reviewed here do not yet give a predictive answer to this. It worth to note that the material input parameters used throughout the models discussed in this Section are not fixed constants but show measurable scatter. Cure kinetics parameters (initial degree of cure, activation energy, reaction order) fitted from replicate DSC tests introduce variability that, once propagated through a coupled cure/heat-transfer simulation, shown to produce a coefficient of variation of around 30% in predicted temperature overshoot for thick-section cure; combined with boundary-condition variability (tool and air temperature, surface heat transfer coefficient), overall cure time variability reaches a coefficient of variation of around 20–22% [70]. Stochastic simulation approaches, coupling a deterministic cure/flow model with Monte Carlo sampling or the Probabilistic Collocation Method, allow this input variability to be propagated through to process outcomes such as cure time, temperature overshoot risk or degree-of-cure uniformity, rather than treating the underlying simulation as if its inputs were known exactly. This matters directly for the residual stress predictions of Sect. 2 and the process windows discussed in Sect. 3, since a deterministic simulation run at nominal parameter values gives no indication of how much confidence to place in the predicted outcome.

## Process modelling of composite manufacturing

### Thermoset composite manufacturing

#### Liquid composite moulding processes

The Liquid Composite Moulding (LCM) families, which includes RTM, VARTM, vacuum infusion and Seemann Composites Resin Infusion Moulding Process (SCRIMP), mainly differ in how fill time is traded against thickness control [71]. LCM processes, such as RTM and VARTM, are well-suited for manufacturing composite components, offering high surface quality and improved structural integrity [72]. In SCRIMP, a high-permeability distribution medium is placed between the reinforcement and the vacuum bag, so the resin can travel quickly across the mould before going through the thickness. This is fast, but it gives poor thickness control, and later variants, such as Light RTM, use a semi-rigid shell instead of the flexible vacuum bag to solve this issue. The flow itself is dual-scale, meaning there is a faster flow between the tows and a slower flow inside them, and this is usually represented in simulation by adding a sink term to the flow equations. Also, the permeability is not constant throughout the process, because compaction and resin uptake keep changing the local fibre volume fraction as the filling proceeds. Moreover, race-tracking along the mould edges or around inserts is still one of the main causes of dry-spot defects [73]. Consequently, modern LCM simulations increasingly seek to couple flow, compaction, permeability evolution and cure kinetics within a unified framework, allowing both filling behaviour and final part quality to be predicted simultaneously.

RTM is widely used for manufacturing complex composite structures, where resin is injected into a closed mould containing dry fibre preforms [74, 75]. Numerical RTM simulations are commonly employed to predict residual stresses and distortions, typically combining sub-models for heat transfer and cure kinetics, flow and compaction, and stress development [59, 76]. Incremental approaches are frequently used, in which cure-dependent elastic properties develop during the curing process, often assuming a linear relationship between the resin modulus and degree of cure, while some models also account for temperature effects [74, 76, 77]. Three-dimensional FE models were employed to utilise these properties to predict stresses and deformations, accounting for thermal strains, cure shrinkage, and tool–part interactions under various bonding or contact conditions. Semi-numerical and unit-cell-based models have been applied by Huang, Gillespie Jr [77] to thick composites, integrating cure kinetics and volumetric shrinkage, and predicting non-uniform curing strains that lead to warpage. Svanberg, Altkvist [65] adopted simplified constitutive models that assume constant properties in both the rubbery and glassy regimes, alongside an abrupt transition at the glass transition temperature. Their models yield reasonable spring-in predictions under specific boundary conditions. More detailed FE simulations were presented by Khoun and Hubert [74] with advanced contact formulations, including frictional interactions governed by Coulomb stick–slip behaviour, to better represent tool–part interaction. These approaches have shown improved agreement with in-situ strain measurements, such as those obtained from embedded fibre Bragg grating (FBG) sensors [78]. Regression-based and reduced-order modelling strategies have been proposed by Dong, Zhang [79] to efficiently predict dimensional variations in complex composite assemblies. These methods, often integrated with FE simulations, significantly reduce computational cost, making them suitable for industrial-scale applications.

**Fig. 6:**
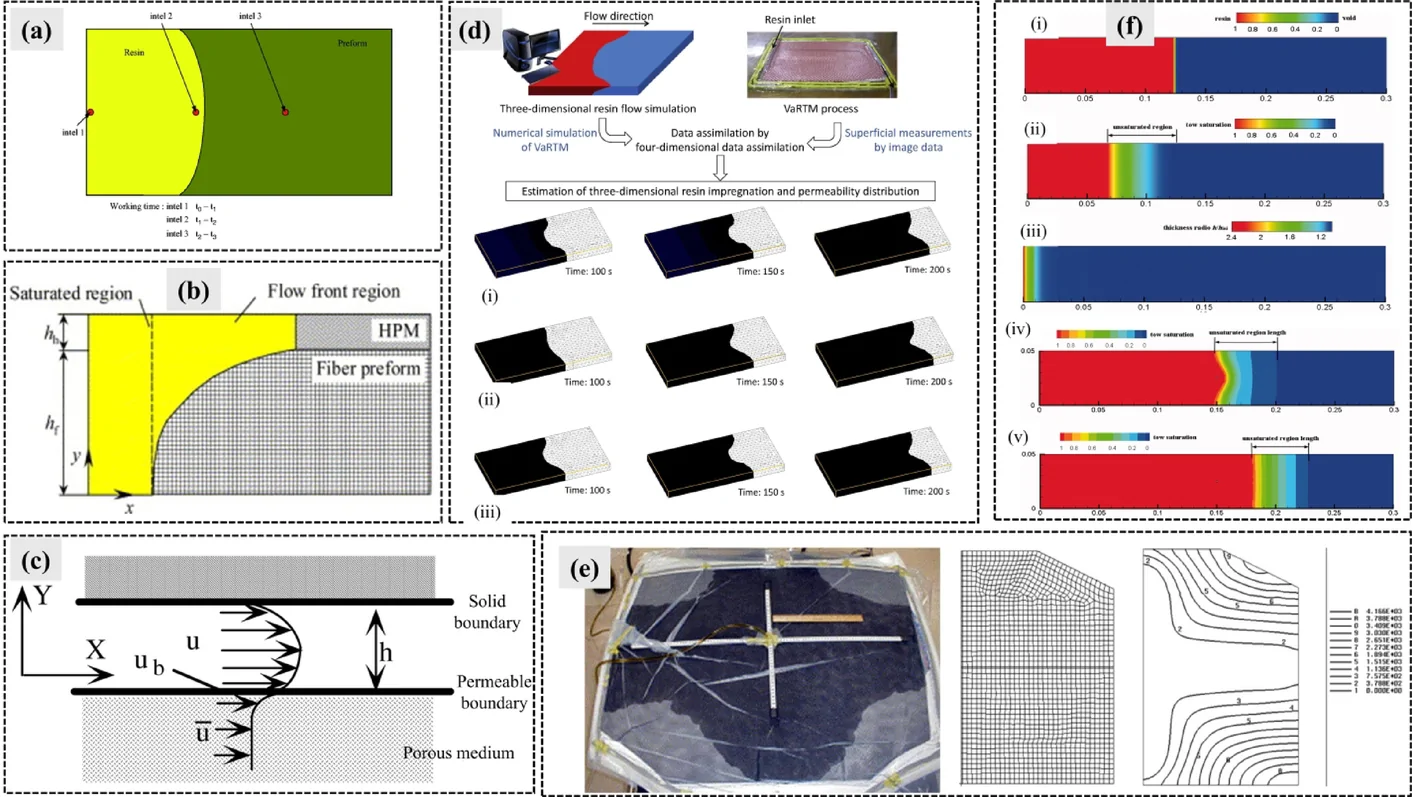
**a** Mould-filling strategy for large composite structures [99], **b** Resin flow in VARTM process [96], **c** Slip boundary at the permeable interface [82], **d** Overview of the flow monitoring technique used, which integrates stochastic simulation of resin flow with visual observation of the flow front location; Resin-filled region during VARTM process. (i) True value, (ii) Estimation by EnSRF, (iii) Estimation by 4DEnSRF [101], **e** VARTM processing and RTM simulation of car hood [104], **(f)** Simulation results of one-dimensional flow, 148 s. (i) Flow front, (ii) saturation distribution and (iii) preform thickness; Shape and length of the unsaturated region in dual-inlet mould filling. (iv) 205 s and (v) 262 s [99]

In contrast to RTM, VARTM significantly reduces tooling costs by utilising a single mould in combination with a vacuum bag for enclosure. This makes it particularly suitable for large-scale composite structures, such as ship hulls and wind turbine blades, where it is regarded as a cost-effective manufacturing approach [80, 81]. The development of flow modelling techniques for the VARTM process has been a progressive area of research spanning over two decades. Han, Jiang [82] pioneered the simulation of the VARTM process by proposing a hybrid 212D (3D model with flow in the thickness direction is neglected) flow model that simultaneously accounted for resin flow from the injection gate and compaction pressure over fibre mats to predict dry spots in complex battleship sections, and the model was successfully validated against ship hull fabrication experiments. Building upon this, Kang, Lee [83] introduced an analytical model incorporating force equilibrium between fibre and resin, enabling mould filling simulation through multiple inlet ports under varying vacuum conditions for naval structures. Song, Loos [84] further advanced the field by systematically evaluating the influence of high permeable medium (HPM) configurations on resin infiltration, incorporating sub-models for resin curing, viscosity variation, and fibre compaction using the FE-CV numerical simulation method. Concurrently, Dai, Pellaton [80] employed the RTMSIM finite element code to investigate the role of HPM layers and inlet channels in sandwich panel fabrication, demonstrating that HPM-based approaches offer both cost and time advantages. Johnson and Pitchumani [85] introduced a novel non-isothermal VARTM simulation framework incorporating induction heating to control resin viscosity and permeability variation. Acheson, Simacek [86] further examined the coupled effects of fibre compaction and tow saturation using non-rigid control volume formulations, revealing that compaction pressure significantly alters the resin pressure profile throughout the infusion process. Chen, Dong [87] addressed computational efficiency by introducing an equivalent permeability approach within the CV/FEM framework, which was validated against automobile part manufacturing, while Hsiao, Devillard [88] developed a genetic algorithm combined with flow simulation to optimise HPM layup and inlet flow arrangements for manufacturing co-cured rib structures. Subsequent research efforts shifted towards refining individual constitutive models and expanding simulation capabilities to more complex geometries and real-world applications. Gokce, Chohra [89] developed an iterative algorithm within the LIMS simulation tool to simultaneously estimate HPM and fibre layup permeability from a single experiment, concluding that HPM permeability varies as a function of the fibre lay-up configuration. Grujicic, Chittajallu [90] utilised a control volume/FE model to simulate non-isothermal preform infiltration and their work demonstrated that heating the tool-plate significantly accelerates infusion rates, with optimal results achieved when 70–80% of the mould is filled at room temperature prior to heating. Dong [91] proposed the equivalent medium method (EMM) for 3D VARTM simulation, reducing computation time by up to 85% while maintaining acceptable accuracy, and further extended this work by applying Design of Experiment (DOE) and Response Surface Method (RSM) to develop a quadratic regression model validated on ship hull and car hood geometries. Lawrence, Frey [92] and Lawrence, Holmes [93] demonstrated the practical applicability of VARTM simulation, first by investigating the effect of embedded impermeable inserts on resin flow and dry spot formation, and later by applying flow simulation tools to the manufacturing of a helicopter pylon assembly upper deck, highlighting the importance of accurate permeability characterisation. Researchers such as Li, Zhang [94] and Robinson and Kosmatka [95] developed a numerical model to investigate the variation in laminate thickness and pressure gradients, accounting for the critical resin bleeding stage that dictates final fibre volume fractions, and validated on a boat deck. Dong [96] further improved computational efficiency through a two-step DOE-coupled CVFE simulation approach, achieving over 99% reduction in computation time. In the following years, Govignon, Verleye [97] developed a 2.5D RI/VARTM simulation using the SimLCM tool for moderately complex geometries, and Ranjbar [98] presented a 3D non-isothermal VARTM model for wind turbine blade manufacturing that optimised inlet port arrangement and process temperatures. Later contributions by Yang, Jin [99] incorporated fibre tow impregnation and layup deformation effects using the VOF method in ANSYS Fluent (see Fig. 6a), while Struzziero [100] applied a multi-objective genetic algorithm optimisation to simultaneously minimise filling time and temperature excess during both filling and curing stages. More recently, Matsuzaki and Shiota [101] introduced a stochastic data assimilation approach using the Karhunen–Loève expansion to accurately estimate permeability fields at low computational cost, enabling precise coupling of thickness variations and real-time visual flow observation, as illustrated in Fig. 6d. Loudad, Saouab [102] addressed the hydro-mechanical coupling between resin flow and fibre compressibility in multilayer VARTM configurations, while Stolz, Fideu [103] utilised COMSOL Multiphysics with extended physics modelling to precisely compute resin flow by coupling thickness variation with preform saturation pressure fields. Figure 6 illustrates representative modelling and monitoring approaches used in VARTM and resin-infusion processes. Figure 6a and b demonstrate typical mould-filling patterns and flow-front evolution during resin infusion, highlighting the importance of inlet configuration, permeability distribution and flow-path optimisation for achieving complete impregnation. Figure 6c shows the slip boundary conditions that may develop at permeable interfaces, which influence local pressure gradients and resin transport behaviour. Figure 6d presents the integration of stochastic simulation with real-time flow-front monitoring, demonstrating how data assimilation techniques can improve permeability estimation and enhance process predictability. Finally, Fig. 6e and 6f illustrate the capability of numerical models to capture resin saturation, thickness evolution and unsaturated flow regions during infusion. Collectively, these examples highlight the ongoing development of VARTM simulations from conventional mould-filling analysis towards coupled flow-monitoring and process-optimisation frameworks that support large-scale composite manufacturing.

The mechanism by which sensing integrates with a running model, rather than simply validating it afterward, is data assimilation: the model’s own prediction is treated as a prior estimate, which is then corrected against the incoming sensor reading using a filtering algorithm, rather than the sensor reading simply being logged for later comparison. Figure 6d, discussed earlier in this review, is a concrete example already present in the literature covered here: an Ensemble Square Root Filter (EnSRF) and its extension 4DEnSRF are used to correct a simulated resin flow front against visual flow observation during VARTM injection, in real time [101]. This is the same underlying principle that later enables the digital twin implementations discussed in this review. Although substantial progress has been achieved in mould-filling simulation, accurate prediction of permeability evolution, race-tracking behaviour and hydro-mechanical coupling between resin flow and fibre compaction remains a major challenge, particularly for large-scale industrial structures.

#### Thermoset cure process modelling

**Fig. 7:**
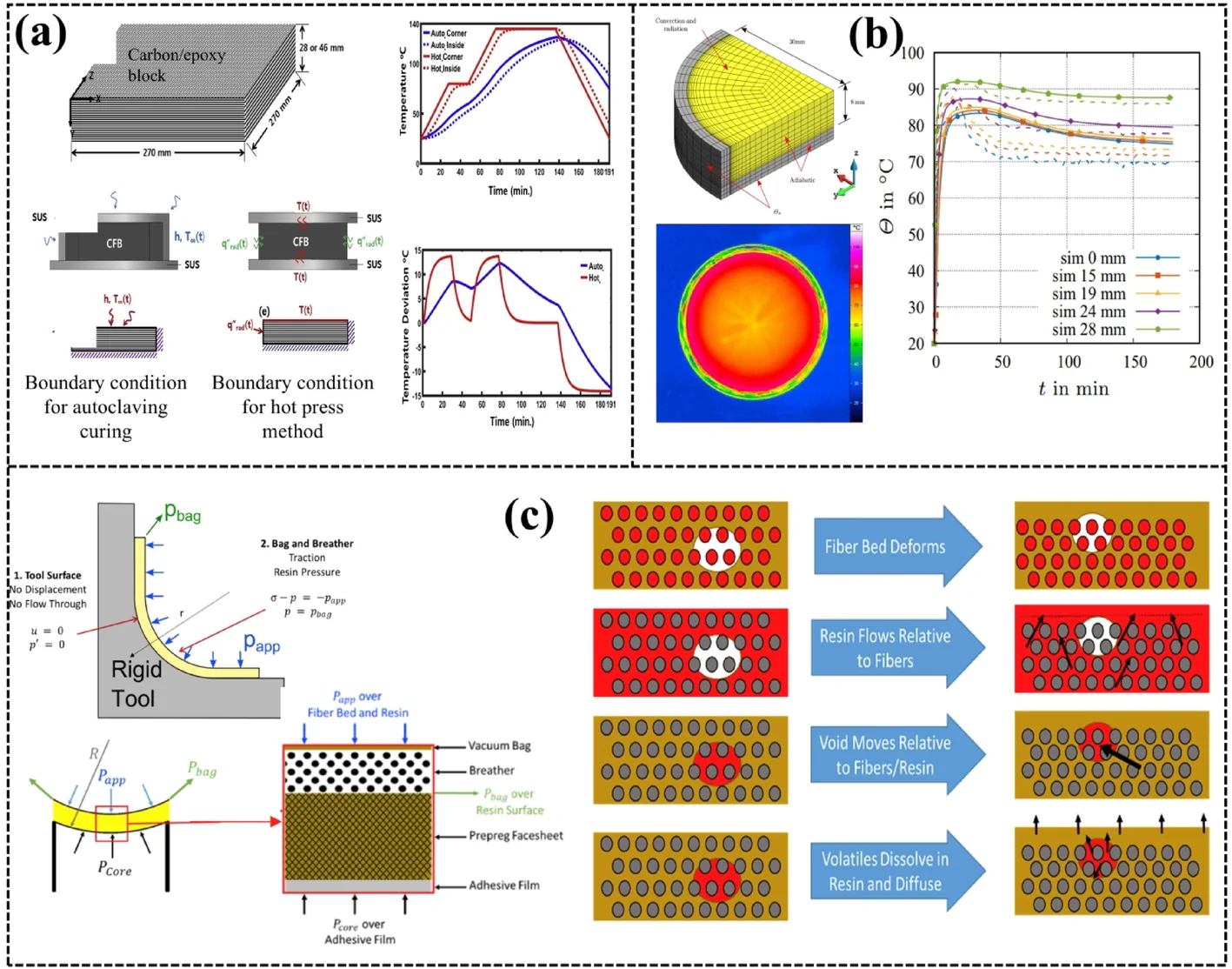
**a** Geometry, setup, and boundary conditions for simulating autoclave and hot press curing processes of carbon/epoxy laminates [107], **b** Finite element simulation of epoxy curing with experimental verification using thermographic measurements [108], **c** Applied boundary conditions and governing physical mechanisms during the initial consolidation stage in autoclave processing [109]

O’Brien and White [105] investigated the EPON resin 862 (Bisphenol F-based) with curing agent EPON W to develop a model for the curing and gelation behaviour of the epoxy system. Ramakrishnan, Zhu [106] presented an experimental analysis and numerical model of a resistive heating approach for a carbon Fibre reinforced epoxy system (EPON 815 with catalyst Epicure 3274). Yoon, Kim [107] presented a computational approach to describe the curing process of three-dimensional carbon fibre epoxy block by finite difference method (FDM) as shown in Fig. 7a. Figure 7a illustrates the geometry, thermal boundary conditions and numerical framework employed to predict temperature and degree-of-cure evolution during carbon/epoxy laminate curing. Simulated results of autoclave curing were validated with the C-scan experimental data. In addition, this approach proposed an optimum cure cycle so that temperature differences in the part could be minimised, resulting in a reduction in the input energy consumption during the cure cycle. It was suggested that energy consumption could be reduced by increasing the heat supply rate before the block temperatures reached the polymerisation point of the resin. The total energy saving was found to be in the range of 7 to 35%, depending upon the method and dimension of the curing block. Leistner, Hartmann [108] performed the finite element modelling of the curing process of epoxy resin, as shown in Fig. 7b. Figure 7b demonstrates the integration of cure-kinetics models with finite-element analysis and experimental thermographic validation, highlighting the importance of accurately capturing exothermic heat generation and spatial temperature distributions. The classical curing model of Kamal-Sourour [4] was calibrated by collecting experimental data on epoxy resin and then implemented in MATLAB. The numerical simulations were validated with the epoxy curing experiment, where a thermal camera performed thermographic measurements at the top surface during curing. Šimáček and Advani [109] described the modelling approach for the first stage of the consolidation process of the autoclave curing. In the first stage, voids were filled, and due to the application of pressure, reinforcements were compressed. Figure 7c shows the physical mechanisms and boundary conditions associated with the initial consolidation stage in autoclave processing, where pressure application, void reduction and reinforcement compaction occur simultaneously. Further, Behzad and Sain [110] developed a transient nonlinear heat transfer coupled with a cure kinetics model to predict the degree of cure and temperature profile during the curing of polymer composites. Joshi, Liu [111] used an FE tool and user subroutines to simulate the resin cure kinetics of a thick graphite/epoxy laminate in a transient heat transfer analysis. However, they did not consider the porosity effects during the cure. Hojjati and Hoa [112] studied a one-dimensional through-thickness curing and consolidation of the thermoset composites. They found that the level of consolidation was non-uniform through the thickness. Yi, Hilton [113] conducted a nonlinear transient heat transfer FE simulation, assuming that the thermal properties of composites are a function of the degree of cure and temperature. Park, Goo [114] established a 3D FE model that can produce an accurate cure-induced temperature profile for composite structures of arbitrary shape with non-uniform autoclave temperature distribution. The cure kinetic parameters were measured using DSC to optimise the cure cycle and acquire a high-quality resin component [115]. Dong, Zhou [116] presented the numerical simulation of the curing process on the composite plate to observe the degree of cure and temperature during the autoclave curing process. A subroutine was developed to couple the heat transfer and cure reaction equations to implement the mathematical model. Simulated temperature evolution in the composite plates coincided with the experimental autoclaving cycle. Results showed that the increase in temperature inside the plate is faster than outside of the plate. Blest, McKee [117] studied the mathematical modelling and simulation of autoclave curing of the multi-layered thermoset composite. Resin flow and cure modelling were based on Darcy’s Law and Stokes equation coupled with a finite difference representation of a convective-diffusion heat transfer equation. Han, An [118] developed a computational fluid dynamics (CFD) model to evaluate the temperature distribution in a mould during autoclave curing with the help of a finite volume method. The deviation between the model and experimental results was 5.9%. Wang, Wang [119] investigated the effects of thermal gradient on autoclave cured laminates through a numerical approach. Results suggested that the uniform curing and lesser deformation of the composite part could be obtained by reducing the thermal gradient. Researchers in [120, 121] proposed a quasi-transient strategy to couple CFD and the FE method for numerical heat transfer simulation in the autoclave. The proposed method reduced the computational effort to 80% compared to the full transient strategy. In addition, the relative temperature error was lower than 4.9 °C (2.3%) compared to the experimental result. Zhang, Xu [23] established a 3D FE model to analyse and simulate the degree of cure and temperature distribution during the curing of the epoxy cast part. The model provided an accurate method for simulating the curing process that allows further insight into the process of curing epoxy resin. It was suggested that this modelling approach could improve the polymer’s mechanical properties by reducing warpage and residual stresses during curing. Collectively, these studies demonstrate that coupled thermal-cure simulations have reached a relatively mature level of development for thermoset composites. Current research is increasingly focused on improving computational efficiency, reducing cure-induced defects in thick laminates, and integrating process simulations with real-time monitoring and control strategies.

### Thermoplastic composite manufacturing

#### Thermoplastic compaction and consolidation

To ensure high‑quality manufacturing of thermoplastic composite components, significant research efforts have been devoted to investigating the development of internal stresses and their resulting deformation behaviour. Karakuzu, Aslan [122] analysed the contributions of multiple factors to residual stress in fibre-reinforced thermoplastic composites, demonstrating that fibre orientation and joining method play dominant roles, while laminate thickness has a minimal effect. For thermoplastics, consolidation quality usually explained through two mechanisms working together: intimate contact, which is the build-up of physical contact area between two rough ply surfaces under pressure and temperature, and healing (also called autohesion), which is the interdiffusion of polymer chains across the interface once contact has been made [123]. Healing cannot happen faster than intimate contact allows, and a dimensionless number has been proposed to show which of the two mechanisms is the limiting one for a given set of process conditions. Void behaviour also connected to the same physics, as interlaminar voids can stay, or even come back, if the interface temperature goes outside the melt or glass-transition range at the wrong point during compaction. Compared with thermoset cure modelling, thermoplastic process simulation remains more challenging because consolidation quality depends on the simultaneous interaction of heat transfer, crystallisation kinetics, intimate contact development, healing and void evolution within relatively short processing times.

**Fig. 8:**
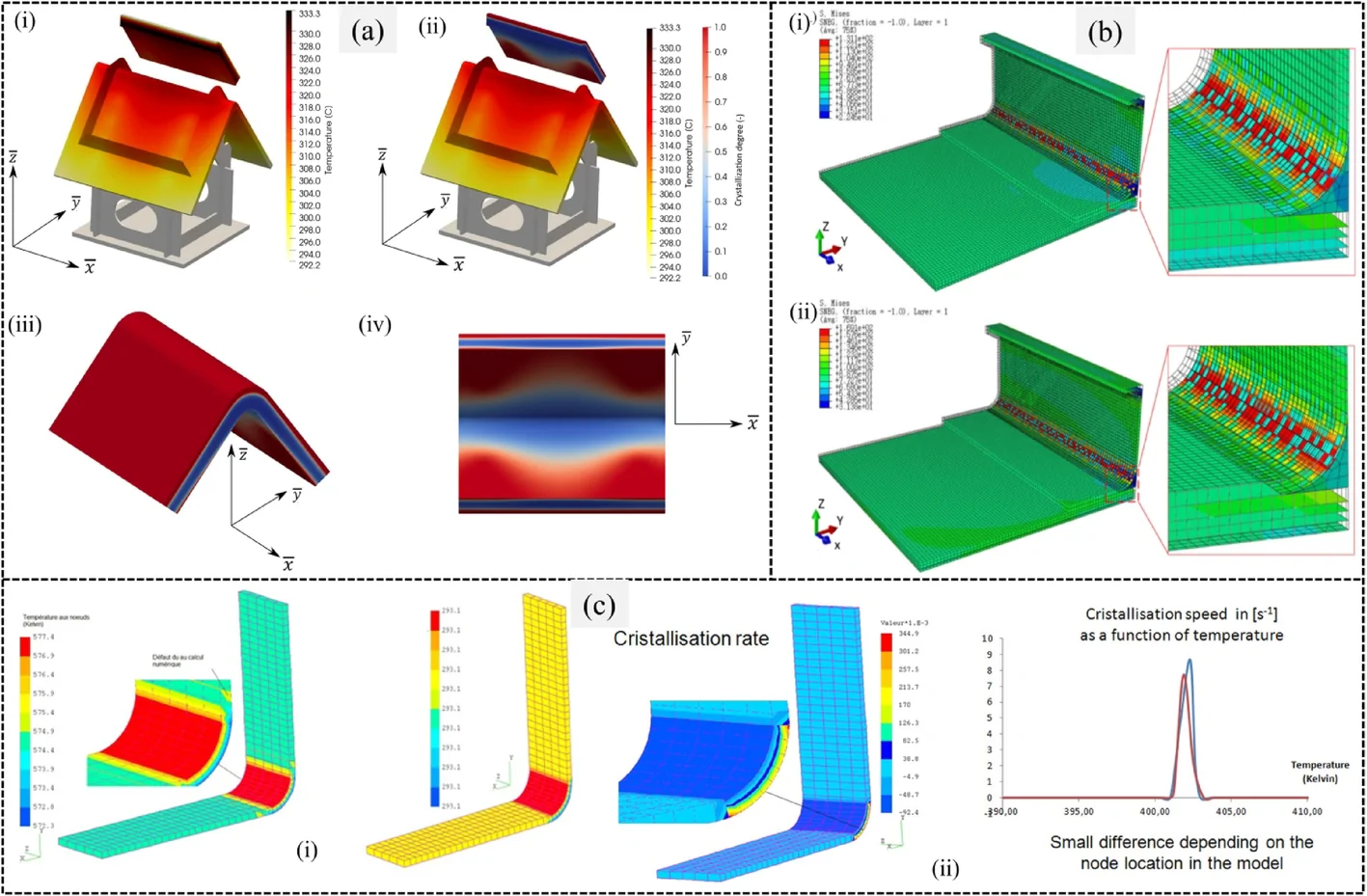
**a** Complex geometry with support and angular features at t = 2.7 h: (i–ii) temperature fields of the mould and half-part; (iii–iv) crystallisation degree distribution within the CFTP component [15], **b** Residual stress contours after cooling: (i) path-dependent model; (ii) crystallinity-based model [11], (c) (i) Temperature evolution at two distinct cooling times; (ii) Predicted crystallisation rate and propagation speed [17]

Péron, Jacquemin [124] proposed a numerical model for predicting residual stresses that accounts for thermal conduction, matrix crystallisation shrinkage, and material mechanical response, and confirmed its accuracy through comparisons with experimental results and classical laminate theory. Denis, Nguyen [15] developed a 3D thermokinetic model in FreeFem + + to simulate the consolidation of thick thermoplastic laminates, coupling transient heat transfer with Nakamura crystallisation kinetics, as shown in Fig. 8a. The research identifies Thermal Contact Resistance (TCR) and mould geometry as critical, non-negligible factors, demonstrating that thick parts exhibit high sensitivity to TCR variations and thermal “fin effects” from mould supports. Experimental validation revealed that steep internal temperature gradients and “keystone effects” in curved geometries drive heterogeneous crystallisation and localised porosities, requiring precisely optimised thermal cycles to ensure material health. Tsukada, Minakuchi 235 utilised Fibre Bragg Grating (FBG) sensors to monitor residual stresses at different through-thickness positions in thick thermoplastic composite laminates. By investigating the effect of annealing, they determined that differences in temperature between the surface and core during cooling result in the formation of thermal residual stresses that persist despite annealing. Dai, Zhan [11] established a resin time-varying modulus model incorporating crystallisation kinetics to simulate the stress evolution and demoulding deformation of thermoplastic composite I-stiffened structures (Fig. 8b). Figure 8b presents residual stress contours from both a path-dependent model and a crystallinity-based model for the same geometry. This is a rare case in the literature reviewed here where two different modelling formulations are applied to identical geometry rather than each being validated only against its own separate dataset, giving anyone implementing either model a direct benchmark to test their own implementation against, rather than relying on experimental validation alone. Online monitoring via integrated Thermocouple and Fibre Bragg Grating (FBG) sensors identified specific stress peaks in the trigonum, skin, and corners (60.7, 84.5, and 169.5 MPa, respectively). By accounting for phase change effects, the Crystallinity model achieved high-fidelity results with error margins of only 9.3% for internal stress and 5.1% for demoulding deformation. Pascon, Bruyneel [17] developed a computational simulation chain in SAMCEF that integrates an original Fast Marching Method (FMM) kinematic draping approach with non-isothermal crystallisation kinetics to model the thermoforming of thermoplastic composites (Fig. 8c). The framework employs fibre-matrix homogenisation to establish temperature-dependent orthotropic constitutive laws and calculates residual stresses by equating mechanical strains with opposing thermal strains under mould-constrained conditions. Application to corner beam geometries successfully predicted process-induced geometric distortions, specifically the “spring-in” effect, while identifying critical non-uniform temperature gradients during the cooling phase. The path‑dependent model proposed by Svanberg and Holmberg [125] characterised the polymer modulus by replacing rate‑related parameters in the viscoelastic formulation with parameters associated with the material state, while treating the glass transition temperature as a step change. According to Wijskamp [126], a solidification point is attained when the relative degree of crystallisation in a thermoplastic reaches approximately 50%, at which point the material behaves elastically as a solid in accordance with continuum mechanics. This transition was identified as a key factor influencing the evolution of the thermoplastic modulus. Brauner, Peters [127] later confirmed these findings and effectively incorporated the concept into deformation studies of L‑shaped structures.

#### Automated fibre placement (AFP)/automated tape placement (ATP) and in-situ thermoplastic consolidation

In automated fibre placement (AFP), the intimate contact and healing mechanisms described above must occur within the residence time at the compaction roller nip point, which for typical CF/PEEK in-situ consolidation, can be as short as a few tens of milliseconds [128]. Consequently, bond quality is highly sensitive to layup speed, compaction pressure and nip-point temperature, since a pressure level sufficient to establish intimate contact may still provide insufficient time for molecular interdiffusion and healing to occur. A surface-topology-based measurement method has recently been used to quantify the degree of intimate contact achieved during placement, providing a means of experimentally assessing a phenomenon that is otherwise difficult to observe directly.

In addition to bonding development, AFP processing is characterised by highly localised and transient thermal cycles generated by laser, infrared or hot-gas heating systems. These rapid heating and cooling rates strongly influence crystallisation behaviour, void evolution and residual stress generation within the deposited laminate. As a result, process simulations typically couple heat transfer, consolidation mechanics, crystallisation kinetics and contact-pressure evolution in order to predict the final quality of the deposited material. Furthermore, the repeated deposition of successive plies introduces thermal accumulation effects that can alter the temperature history experienced by previously deposited layers, thereby affecting local crystallinity and bonding quality throughout the structure. In addition to consolidation and bonding behaviour, recent AFP modelling efforts have increasingly focused on the prediction of manufacturing defects such as voids, gaps, overlaps, fibre waviness and local thickness variations. These defects can significantly influence the mechanical performance, dimensional accuracy and long-term reliability of the final component. Consequently, advanced simulation frameworks increasingly seek to integrate defect prediction with thermal, mechanical and material-state evolution models to support process optimisation and quality assurance. Process modelling of AFP therefore focuses on predicting temperature evolution, degree of consolidation, bonding quality, crystallinity development, void content and residual stress formation at the nip point and within the growing laminate. Furthermore, the growing adoption of infrared thermography, laser-based inspection systems and embedded sensing technologies has created opportunities for coupling process simulations with real-time monitoring data. Such developments are expected to support digital-twin-assisted manufacturing and adaptive process-control strategies in future AFP production environments. Despite significant advances in local-scale modelling, validated simulation frameworks capable of accurately predicting the cumulative effects of multiple deposition passes, layer-by-layer thermal interactions and process-induced deformation across large structural components remain limited and continue to represent an important research challenge.

#### Welding and joining of thermoplastic composites

Resistance, induction and ultrasonic welding are among the most widely used techniques for joining thermoplastic composites, each generating heat at the bonding interface through a distinct mechanism. In resistance welding, electrical current is passed through an embedded heating element, producing Joule heating and local melting of the thermoplastic matrix. Although this approach offers good process control and is suitable for large structural assemblies, the heating element remains within the joint and may influence long-term performance [129]. Induction welding generates heat through electromagnetic induction, whereby eddy currents are induced within the conductive laminate or a susceptor, enabling rapid, non-contact heating without a permanent heating element. Ultrasonic welding generates heat through high-frequency mechanical vibrations and has recently progressed from spot welding to continuous welding of larger structural components [130, 131].

From a physical perspective, all three methods can be regarded as accelerated forms of thermoplastic consolidation. Heat generation promotes matrix melting, intimate contact, resin squeeze flow and molecular interdiffusion at the interface, while subsequent cooling governs crystallisation, residual stress development and final joint quality. Consequently, weld performance depends strongly on thermal history, consolidation pressure and cooling conditions. Process models therefore couple heat transfer, fluid flow, healing, contact evolution and crystallisation kinetics to predict bond formation and structural performance. For induction and resistance welding, recent multiphysics models have incorporated electromagnetic, thermal and mechanical effects to optimise process parameters and improve heating uniformity [130, 132].

Ultrasonic welding has attracted considerable attention because of its short cycle times and high energy efficiency. Modelling studies generally focus on heat generation, resin flow and interface evolution, with weld quality being particularly sensitive to vibration amplitude, welding pressure, weld time and energy-director geometry [133]. Despite significant progress, accurately predicting the coupled effects of heat generation, consolidation, healing, crystallisation, residual stresses and defect formation remains a major challenge. Future developments are expected to focus on validated multiphysics models integrated with in-situ monitoring and digital-twin technologies to improve weld-quality prediction and process optimisations.

### Other common manufacturing processes

#### Draping/forming and preforming process

Draping simulations mostly built using shell finite elements, and majorly they track the mean-surface deformation of the fabric, mainly the in-plane shear angle and the onset of wrinkling [134]. The through-thickness kinematics could not be resolved unless the reinforcement is thick, in which case a full 3D simulation becomes necessary. Moreover, it was found in the literature that the shear locking angle alone is not a reliable predictor of wrinkling, since wrinkling depends on tensile, in-plane shear and bending stiffness acting together with the boundary conditions, and not on the shear angle by itself [135]. For multi-layered preforms this becomes more complicated, as neighbouring plies at different orientations can develop local zones of fibre compression, which can trigger wrinkles even where a single ply would drape without issue. Coupled meso-macro approaches have also been used for this, where a macro-scale draping simulation gives the boundary conditions for a more detailed meso-scale model of a local patch, this way, yarn slippage and distortion around double-curved features such as mould corners can be captured without having to run the full simulation at yarn level [134]. Because fibre orientation obtained during draping directly influences local stiffness, permeability and defect formation, draping simulations increasingly form the first stage of integrated process-structure-performance modelling frameworks.

#### Pultrusion and filament windings

Pultrusion and filament winding are continuous composite manufacturing processes that present distinct modelling challenges compared with mould-based processes such as RTM and autoclave curing. In both cases, process modelling aims to predict material state evolution, residual stress development and dimensional stability while accounting for the strong coupling between thermal, chemical and mechanical phenomena. Pultrusion models generally comprise a set of coupled sub-models describing cure or crystallisation kinetics, heat transfer, resin flow, pulling force and stress-strain evolution as the material continuously moves through a heated die [136]. Unlike RTM, where the material remains stationary during cure, pultrusion requires the governing equations to be solved in a moving reference frame. The process is highly sensitive to pulling speed, die temperature distribution and resin reaction kinetics, as these parameters control cure development, internal temperature gradients and the resulting residual stresses. More recently, process-modelling approaches have been extended to curved pultruded profiles, in which partially cured sections pass through pre-forming stages before complete curing, introducing additional geometric and mechanical complexities [137].

Filament winding is widely used for the manufacture of cylindrical and pressure-containing structures, including pipes, tubes and Type IV hydrogen storage vessels. Process modelling typically focuses on predicting fibre trajectories, winding angle distribution, resin curing behaviour and residual stress development during winding and subsequent curing stages. Winding tension is recognised as one of the most influential process parameters. Several studies have shown that mechanical performance improves with increasing tension up to an optimum level; however, excessive tension can elevate residual stresses, induce fibre damage and reduce structural reliability [138]. For this reason, process simulations are increasingly employed to optimise winding parameters and achieve a balance between consolidation quality, dimensional accuracy and mechanical performance.

Despite considerable advancements in both pultrusion and filament-winding simulations, challenges remain in accurately capturing process-induced defects, material-property evolution and residual stress accumulation, particularly for complex geometries and thick composite structures. Future developments are expected to focus on improved multiphysics coupling, integration with in-situ process monitoring and computationally efficient process-structure-performance simulation frameworks.

### Residual stress and distortion prediction

**Fig. 9:**
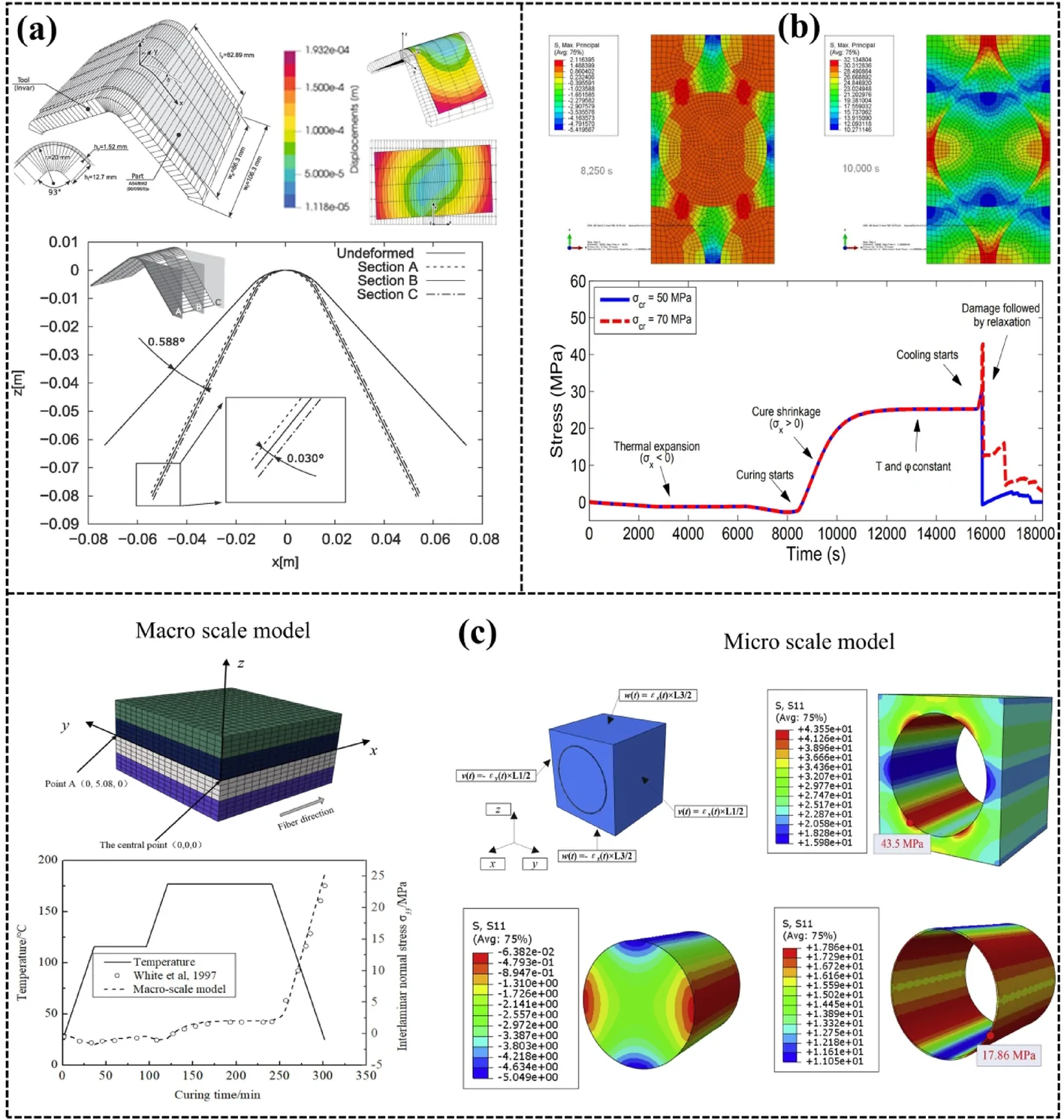
**a** Final deformation of the L-shaped composite structure after completion of the cooling phase [147], **b** Stress development throughout the curing process in a 3D hexagonal representative volume element [148], **c** Three-dimensional multiscale thermo-mechanical simulation of residual stress in fibre-reinforced composites [24]

The process simulations discussed in the previous sections ultimately aim to predict manufacturing-induced residual stresses and dimensional distortions, which represent the most direct link between processing conditions and final component performance. Designing composite structures is inherently complex, as it requires determining material characteristics and mechanical responses that meet predefined performance requirements. Song, Waas [139] observed that the overall properties of 2D triaxial braided carbon fibre composites did not match with attributes based on the individual element properties. This discrepancy was called matrix alterations caused by curing in the presence of reinforcements or fibres. The variations were generated due to an uneven thermal coefficient of expansion, cure shrinkage of the epoxy, and thermal gradients, which resulted in the non-uniform degrees of cure across the volume of the matrix. Therefore, stress which was present after the completion of the cure cycle might reduce the load limit. Such stresses were responsible for the beginning of the inelastic behaviour and led to the failure of the components. Because of the relevance of epoxy resin in structural applications, the determination of residual stresses has received interest in the literature. Such residual stress can be experimentally measured with the help of X-ray diffraction (XRD) [140, 141], strain gauges [142], Raman spectroscopy, and ultrasonic acoustics [143]. Among them, the XRD measurement technique effectively evaluates the residual stress in crystalline materials. Izumi, Kakara [144] used the X-ray diffraction method to study the in-situ residual stress of phenolic resin and copper foil composite during curing. The residual stress at the interface of the resin-copper in the composite was evaluated using the lattice strain of the copper plane. In the semi-cured sample, the copper residual stress was 60 MPa in compression; in the cured sample, it was a compression stress with an amplitude of 114 MPa. Ifju, Niu [145] introduced a unique procedure called the cure referencing method for measuring the residual strain associated with residual stress on the polymer composite surface. Benedikt, Kumosa [146] evaluated the residual thermal stress in the unidirectional graphite fibre polyimide (PMR-15) having embedded crystalline particles (i.e., aluminium and silver inclusions) by XRD measurement techniques. Stresses in the composite samples were observed in the XRD by calculating the lattice space using Bragg’s law. The average magnitude of residual stress components (σ_11_ and σ_22_) on the composite sample was 50 MPa and 29 MPa, respectively. Methodologies are used to model and describe the evolution of stress during the curing of epoxy. Zappino, Zobeiry [147] presented a model for predicting the process-induced deformation (spring in angle) and residual stress of the L-shaped component during curing. The displacement field during cooling time and cross-section deformation are shown in Fig. 9a. D’Mello, Maiaru [148] introduced the computational model to analyse the cure cycle effect on the FRP composites mechanical response. An RVE-based model contains the hexagonal pattern of the fibres that were analysed considering several cure cycles and tailored to reduce the damage during the curing. The contour plot of stress and stress with respect to time graph with different aspects during cure is shown in Fig. 9b. Yuan, Wang [24] studied the residual stress generated during the curing of polymer composites. The author proposed a multiscale RVE-based model, which incorporates the thermochemical and residual model during the curing process. This model was tested with three different arrangements of fibre, i.e., square array, hexagonal array, and diamond array under cure cycle. Model results found that the diamond fibre arrangement model has the maximum micro residual stress among the hexagonal and square. The micro-scale stress plot and macro-scale contour plot of stress are shown in Fig. 9c.

Kappel, Stefaniak [149] presented a semi-analytical simulation approach to predict the induced warpage deformation of carbon fibre polymer composites processed on n autoclave. Warpage deformation was modelled by applying a modified thermal expansion parameter (which depended on the specimen dimensions) at tool-part contact area of the laminate. Plepys [150, 151] described stress evolution and elastic characteristics as a function of cure using incremental elasticity. They postulated that the relation between the stress and strain increment was isotropic and linearly elastic with a varying modulus at each time increment of cure. Bogetti and Gillespie Jr [46] quantified stress relaxation and chemical hardening using a linear mixing algorithm. This algorithm was based on the extent of curing and the moduli of uncured and fully cured resin [152, 153]., and [154] used a linear viscoelastic material model, which was implemented to assess residual stress in polymers and composites. The evolution of cure-dependent (and hence, time-dependent) material characteristics was analysed using integral constitutive equations. Zhang, Zhang [155] established an improved viscoelastic constitutive model to estimate the residual stress in polymer matrix composites while curing. Yuan, Wang [24] proposed a multiscale model for predicting the micro residual stress using periodic boundary conditions (PBC) while curing Fibre reinforced composites. It was assumed that the temperature and degree of cure would remain constant within each finite element at the macro-scale. Adolf and Chambers [25] suggested another model based on non-linear viscoelasticity. However, due to the numerous parameters, such a framework needs substantial validation and experimental examination [156], which is lacking.

Process-simulation predictions are validated against experimental measurements through a combination of material-level and structural-level techniques. At the material level, Differential Scanning Calorimetry (DSC) provides cure-kinetics and heat-of-reaction data used to calibrate Eqs. 11–16, while Dynamic Mechanical Analysis (DMA) characterises the temperature- and frequency-dependent modulus data required to populate the Prony series in Eqs. 32–36 [4]. At the structural level, embedded Fibre Bragg Grating (FBG) sensors provide in-situ strain measurements and, indirectly, cure-state monitoring throughout the cure cycle by tracking wavelength shifts caused by combined thermal and mechanical strains, although separating these two contributions remains a recognised challenge for FBG-based validation [157]. Digital Image Correlation (DIC) enables full-field measurement of surface strain and deformation and has seen increasing use for validating in-process deformation during automated manufacturing routes such as AFP and stamp forming, where full-field measurements are required to capture non-uniform strain development across the tool surface [158]. Thermocouples remain the simplest and most widely used approach for validating thermal predictions, although they provide measurements only at discrete locations rather than over a full field. For residual-stress assessment, the incremental hole-drilling method is the only technique supported by an established ASTM standard (ASTM E837), making it one of the most commonly reported validation approaches; however, it is generally limited to near-surface stress measurements. Slitting and curvature methods are often employed as complementary techniques for through-thickness stress evaluation and overall distortion assessment, respectively [159]. Finally, dimensional inspection using a Coordinate Measuring Machine (CMM) or three-dimensional scanning remains the most direct, but least diagnostic, validation method because it captures the combined effects of all process-induced mechanisms without distinguishing their individual contributions.

Across the process simulation studies reviewed in this section, RTM receives substantially more attention than thermoplastic consolidation, with autoclave curing occupying an intermediate position between the two, reflecting the relative industrial maturity of these processes; pultrusion, by contrast, is covered only in passing through a single reference [35] rather than as a dedicated subsection. Validation rigour also varies across the studies reviewed, with at least one proposed viscoelastic framework explicitly noted as lacking the experimental validation its complexity would require [25, 156].

While experimental agreement is frequently reported in the literature, the level of validation rigour varies considerably between studies. Validation based solely on temperature histories measured using thermocouples or on final part distortion provides only partial confidence because different model assumptions may produce similar macroscopic outcomes. More rigorous validation requires verification at multiple scales and across multiple physical fields. For example, cure kinetics and crystallisation models should first be calibrated using DSC measurements, viscoelastic and stiffness evolution should be validated using DMA or mechanical testing, and process-scale predictions should subsequently be compared with in-situ strain measurements, residual stress measurements, and final dimensional inspection. Such hierarchical validation provides greater confidence that the underlying physics are being correctly represented rather than merely reproducing a final observable response. Despite significant progress, the composite process-modelling community still lacks widely adopted benchmark problems equivalent to those available in some other computational mechanics disciplines. Validation studies continue to rely predominantly on process-specific geometries, materials and experimental setups, making direct comparison between modelling approaches difficult. The development of standardised benchmark experiments incorporating thermal history, degree of cure or crystallinity, residual stress and final distortion measurements would substantially improve model comparison, validation consistency and industrial confidence in process-simulation methodologies.

## Review of multiphysics modelling approaches of composite processing

### Molecular dynamics modelling

Within the overarching framework of computational process modelling, accurately representing material behaviour across multiple length scales remains a fundamental challenge. At the atomistic level, Molecular Dynamics (MD) simulation serves as a powerful process modelling tool for characterising the structural architecture and constituent properties of materials [160]. Rather than depending on empirically derived constitutive formulations, MD-based process modelling harnesses Newton’s second law of motion to yield a granular, physics-driven understanding of failure initiation, progressive damage evolution, and underlying deformation mechanisms [161]. A significant advantage of this modelling approach is that the resulting constitutive responses inherently embed atomistic-level information, thereby eliminating the need for phenomenological assumptions commonly adopted in higher-scale process models [162]. Once a representative molecular configuration is successfully constructed within the modelling framework, extracting the mechanical response via MD becomes computationally tractable. In the process modelling paradigm, MD has been extensively employed to investigate the mechanical behaviour of a broad spectrum of composite systems and their individual constituents, encompassing graphene [163], epoxy resins [163], polymer–graphene nanocomposites [164–166], and polymer–carbon nanotube (CNT) systems [167, 168]. Central to all such modelling investigations was the development of a physically representative molecular model, which constitutes a critical prerequisite for accurately capturing material constitutive behaviour. Considerable research efforts have therefore been directed towards establishing robust MD-based process modelling protocols for diverse material systems, including silica glass [169], covalently cross-linked epoxy network structures [170], and epoxy–silica interfacial assemblies [171]. Although MD simulations inherently operate at strain rates several orders of magnitude beyond those attainable under conventional experimental conditions, they nonetheless yield invaluable atomistic-scale insights that directly inform and enrich higher-level process modelling frameworks governing constitutive responses, deformation pathways, and failure mechanisms.

**Fig. 10:**
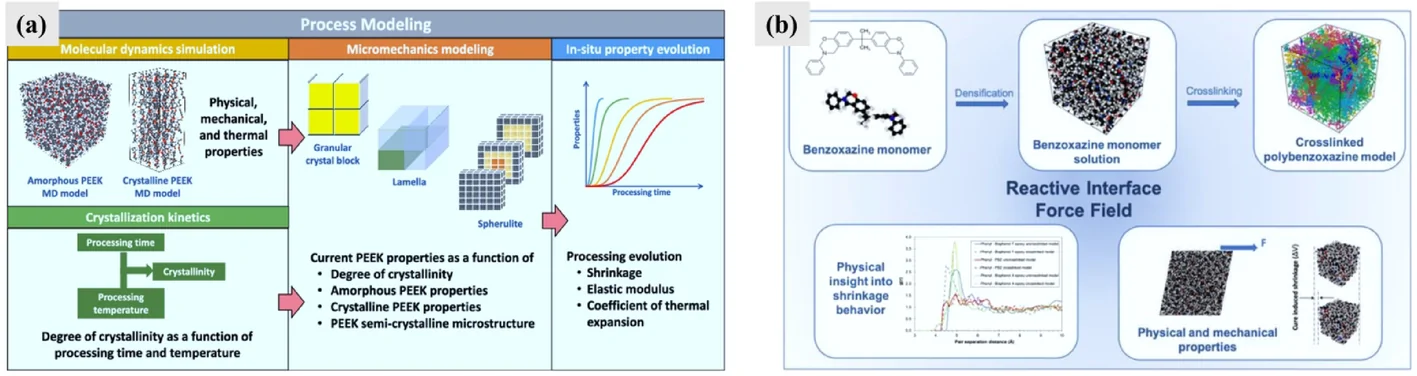
**a** MD-based multiscale modelling of PEEK [176], **b** Molecular modelling of PBZ resin by MD simulation [177]

Within process modelling, multiple works demonstrate that MD simulations can directly provide constitutive parameters for higher-scale modelling. Yang, Ghosh [172] successfully retrieved elastic-plastic constitutive parameters from thin-film MD simulations conducted at a defined strain rate, establishing a direct link between atomistic data and process-modelling constitutive inputs. In a comparable manner, Li, Tang [173] identified and calibrated constitutive parameters for an elastomeric material system derived from MD simulations performed across strain rates of a similar order of magnitude, further reinforcing the viability of MD-informed process modelling approaches. Extending this methodology to rate-dependent material behaviour, Zhao, Ries [174] executed a series of MD simulations spanning a broad range of strain rates to systematically develop viscoelastic constitutive models transferable to the continuum process modelling scale. Beyond direct parameter extraction, researchers have also pursued strategies to address the inherent strain-rate disparity between MD simulations and experimentally relevant loading conditions in process modelling contexts. These efforts have involved the formulation of physically motivated scaling laws and rate-temperature equivalence frameworks to extrapolate atomistically derived material properties into quasi-static regimes applicable to practical process modelling scenarios. Exemplifying this approach, Vu-Bac, Bessa [175] developed a comprehensive multiscale process modelling framework that systematically incorporates constitutive modelling assumptions and MD-calibrated parameters, effectively bridging the atomistic and continuum length scales within a unified modelling architecture. Kashmari, Al Mahmud [176] developed a multiscale process modelling framework for semicrystalline Polyether Ether Ketone (PEEK), integrating three hierarchical computational tiers, i.e. MD simulation, micromechanical modelling via the Multiscale Generalised Method of Cells (MSGMC), and dual-mechanism parallel Avrami crystallisation kinetics to predict thermomechanical property evolution during annealing at 306–328 °C (Fig. 10a). MD simulations predicted amorphous PEEK Young’s modulus of 2.87 ± 0.40 GPa and crystalline axial modulus of 142.14 ± 7.40 GPa, with a phenomenological viscoelastic mapping function employed to bridge the MD strain rate of 2 × 10⁸ s^− 1^ to laboratory-scale conditions. Results demonstrated that lower annealing temperatures produced faster volumetric shrinkage and more rapid evolution of elastic and thermal properties towards crystalline limiting values, whereas higher processing temperatures slowed crystallisation, allowing greater time for residual stress relaxation in PEEK composite laminates. Gaikwad, Krieg [176] employed MD simulation with the Reactive Interface Force Field (IFF-R) to predict the physical, mechanical, and thermal properties of bisphenol-A benzoxazine (Ba-BZ) resin as a function of cross-linking density (10–70%), identifying a gel point at 40–45% and predicting a fully cross-linked Young’s modulus of 5.58 ± 0.10 GPa, yield strength of 131.98 ± 3.95 MPa, and glass transition temperature of 195.21 ± 10.89 °C, all validated against experimental measurements. Radial distribution function analysis revealed that the near-zero volumetric cure shrinkage of PBZ originates from negligible phenyl group rearrangement and invariant nitrogen atom charge (− 0.165) during cross-linking, providing atomistically grounded process modelling inputs for residual stress prediction in polybenzoxazine (PBZ) composite manufacturing (Fig. 10b).

In the context of multi-scale process modelling, MD simulation holds considerable potential for incorporation into macro-scale mechanical simulation frameworks, where it can operate both as an atomistically informed constitutive model and as a concurrent failure evaluation mechanism across varying process modelling length scales. Despite this potential, the prohibitively high computational demand associated with MD remains a primary bottleneck, significantly constraining its practical deployment for instantaneous, on-the-fly characterisation of material mechanical responses within larger process modelling simulation workflows. Consequently, the formulation of surrogate constitutive models capable of accurately reproducing MD-derived mechanical response data presents a highly advantageous strategy for facilitating computationally efficient, real-time predictions within process modelling pipelines. To this end, a range of methodological approaches has been proposed for constructing physics-informed constitutive models grounded in molecular simulation data, specifically tailored to meet the computational demands of process modelling applications. Prominent strategies include the direct calibration of constitutive model parameters against MD-generated datasets [178], as well as the employment of MD simulations in a corrective capacity, wherein atomistic results are leveraged to offset the inherent inaccuracies and oversimplifications introduced by the governing assumptions of conventional constitutive frameworks embedded within process modelling routines [179]. Despite its ability to provide fundamental insight into molecular-scale mechanisms, the application of MD remains limited by computational cost and accessible timescales, restricting its direct application to industrial-scale process simulations.

### Micro and macro scale modelling

Most of the predictive capability of multiscale composite process modelling relies on Representative Volume Elements (RVEs), which provide a microscale representation of the fibre-matrix architecture. By resolving local constituent behaviour, RVEs enable the determination of homogenised thermo-mechanical properties that serve as inputs for structural-scale FE simulations [180, 181]. This scale-bridging machinery underlies several of the models discussed elsewhere in this review, including the effective CTE and modulus values used in the residual stress predictions in Sect. 2.3 and the crystallinity-dependent property evolution discussed in Sect. 2.1.4.

**Fig. 11a:**
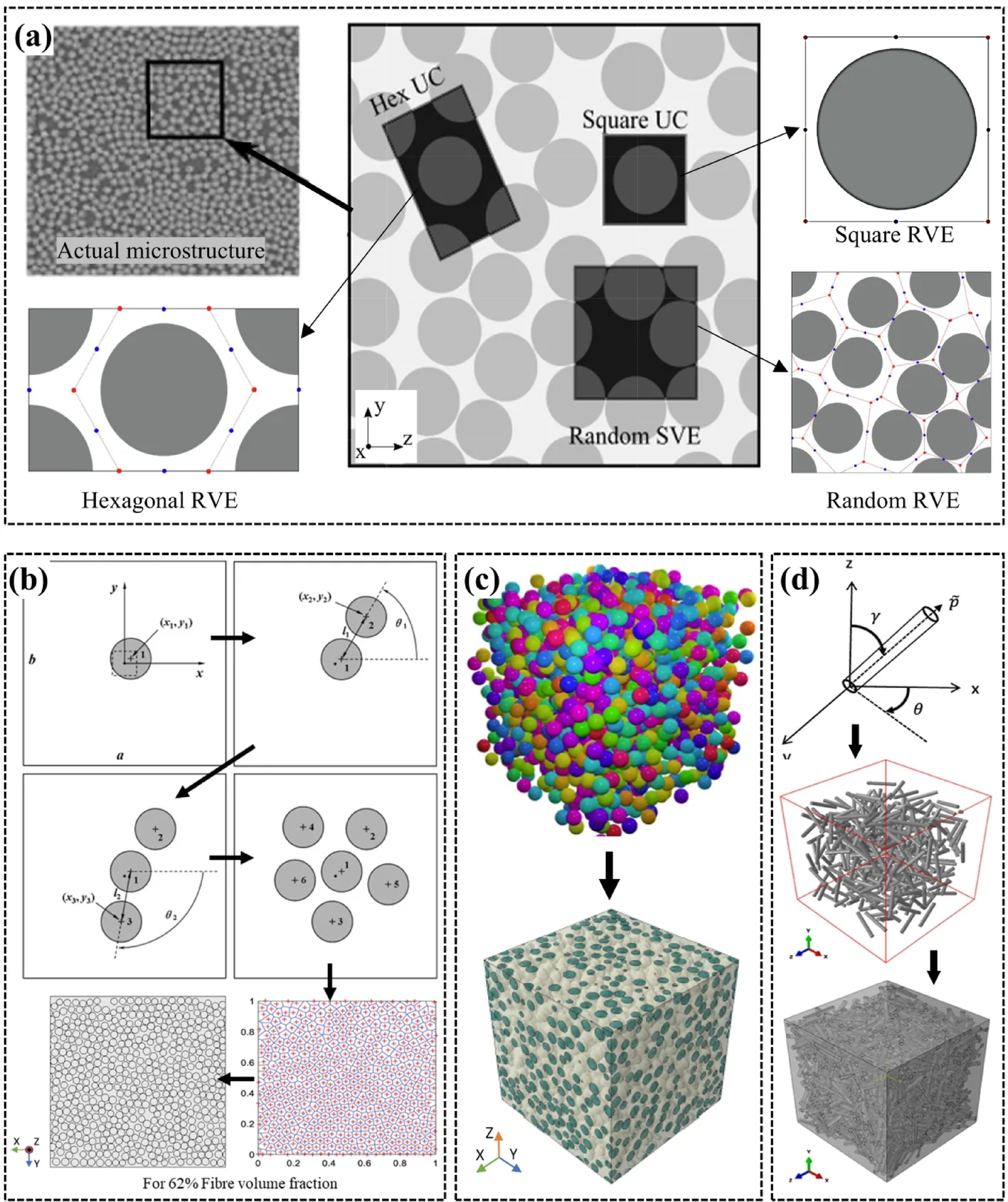
Micromechanical RVE configurations (hexagonal, square, and random) for unidirectional composite laminae [182], **b** Random sequential expansion method for unidirectional RVE construction [183], **c** Periodically arranged random spherical particle RVE via MD simulation [181], **d** RVE with randomly distributed cylindrical inclusions [185]

RVE generation is an important step in the micromechanics or multiscale approach to composite modelling. A simplified approach for modelling the unidirectional RVE, considering the square and hexagonal packing of the fibres, was given by Balasubramani, Zhang [182], as shown in Fig. 11a. In order to narrow down the focus of the investigation, it was assumed that the fibres had a circular form and a consistent diameter within each individual RVE. This assumption disregards any variations in the location of the fibre centroid along the path of the fibre. Yang, Yan [183] presented a novel method for creating a random arrangement of fibres in a 2D cross-section of fibre-reinforced composites with a high fibre volume fraction. The authors termed the algorithm ‘random sequential expansion’ (RSE). The representation of the RSE method and 62% fibre volume fraction RVE is shown in Fig. 11b. The RSE model was built based on the hard-core model (which considered the fibres as a collection of non-overlapping discs, with their centres randomly distributed within a specified area) but surpasses the jamming limit (i.e. fibre distribution with volume fractions more than 50% are rarely produced by it) associated with the model.

Catalanotti [184] created an algorithm to generate the uniform distribution of spherical and circular particles to generate the particle-based RVE. This algorithm can generate RVE, including random spherical and circular particles with any volume fraction from 0 to 0.74 and 0.91, respectively. Li, Sharifpour [181] developed an algorithm to rapidly generate the periodic RVE for the random distribution of spherical particle-reinforced composites as shown in Fig. 11c. This algorithm was based on event-driven molecular dynamics. The generated RVE was compared with available microstructural and optical images of composite materials and showed excellent statistical correlation. It was suggested that the proposed algorithm could be used for the accurate evaluation of the mechanical properties of composite systems. Islam, Tudryn [185] presented a computational approach for creating microstructure models of random cylindrical short-fibre composites, as presented in Fig. 11d. These inclusions have a large volume percentage and a wide variation of aspect ratios. The presented method was based on the random sequential adsorption (RSA) algorithm implemented in MATLAB. Zhang and Yan [180] presented a novel method to generate the random spatial distribution of fibres in transverse cross-sections by random disturbance and elastic collision algorithm. Cai, Ye [236] presented a two-step novel procedure to generate and evaluate the mechanical properties of the random Fibre reinforced composites by considering primary micropores inside. The first micro-CT experiment was performed on a composite sample to detect the micro-scale morphology, i.e. the size of pores, location and distribution of pores and fibres, and then based on the knowledge of the location, a three-dimensional finite volume directly averaging micromechanics were used to establish the microscale model. It was found that around 90% of pores were spherical in shape.

**Fig. 12:**
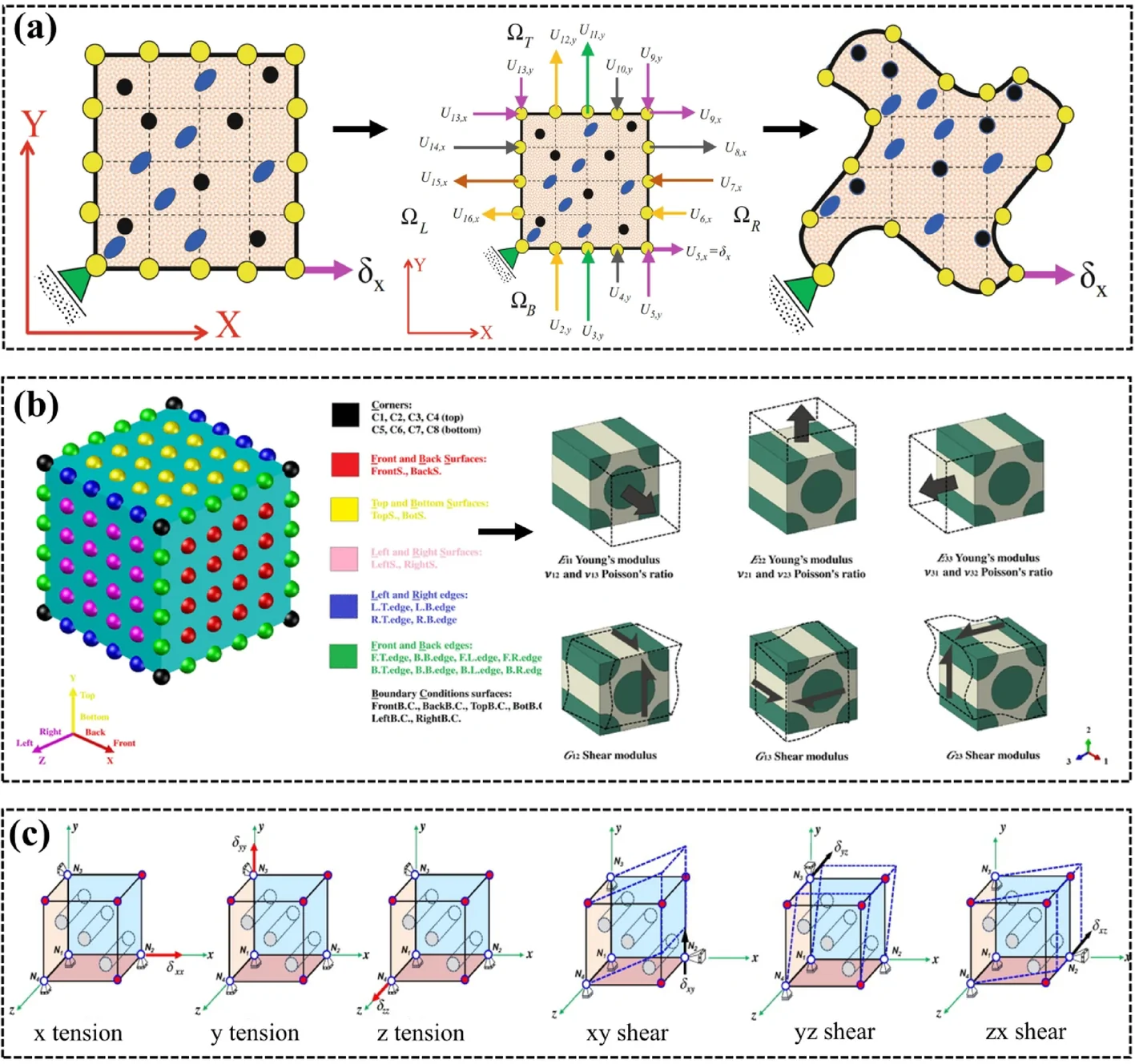
**a** Application of periodic boundary conditions to a 2D RVE and its resulting deformation profile [194], **b** Periodic boundary condition implementation on a three-dimensional RVE [187], **c** A 3D fibre composite RVE subjected to three uniaxial and three shear loading modes under periodic boundary conditions for finite element homogenisation [190]

Periodic boundary conditions are typically applied to the RVE to avoid edge effects and ensure the extracted properties are representative of an infinite periodic microstructure [186, 187]. Where the coupling between scales must stay active throughout the simulation, rather than being computed once and passed up. FE² approaches solve a full micromechanical RVE problem at every integration point of the macroscale model, at correspondingly higher computational cost, and this restricts current FE² applications to relatively small structural domains or reduced-order surrogate versions of the RVE response [188–190]. Past research [191–193] have shown that better prediction of the effective properties was achieved by imposing the periodic boundary conditions (PBCs) on the repetitive unit cell. Okereke and Keates [194] presented the implementation of the periodic boundary condition on the 2D RVE. PBC implementation was by connecting the surface nodes of the RVE. The connection of the surface nodes and the deformed surface in the PBC is shown in Fig. 12a. 3D RVE PBC implementation was demonstrated by Omairey, Dunning [187], where the canonical equation that connected the surface node, edge nodes and corner nodes was presented. The categorization of surface nodes, corners and edges are shown in Fig. 12b. Wang, Zhang [186] presented a novel approach to impose the periodic boundary condition for the three-dimensional braided composite-based RVE model by introducing the radial point interpolation method (RPIM). Periodic boundary conditions were checked with both periodic and non-periodic mesh. This method was checked with six kinds of monotonic loading conditions. The RPIM method was found effective for both periodic and non-periodic mesh.

Homogenisation becomes an important aspect of the modelling of composites, which predicts the effective properties. Until a few decades ago, the technique to homogenise composites and estimate their effective mechanical and thermal properties was to conduct experimental testing on existing test samples [195, 196]. Gradually, the analytical or numerical prediction of the mechanical characteristics of composites became an alternative to the expensive experimental setups [197, 198]. Analytical and numericalmethodologies such as Mori–Tanaka (M–T) method [188], mean-field homogenisation model (single step and two-step) [189], RVE base FE homogenisation method [199], for predicting the effective mechanical and thermal characteristics of composites were developed. Okereke and Akpoyomare [190] presented the FE homogenisation of the RVE by applying 6 independent loads (3 tensile and 3 shear) to obtain the effective mechanical properties of the unidirectional composite material, as shown in Fig. 12c. Tian, Qi [199] introduced the RVE-based finite element homogenisation method with periodic boundary conditions to evaluate the effective mechanical properties of composites with random and complicated microstructures. The homogenised effective elastic properties of the composites with complicated microstructure and periodic boundary conditions were compared with the Halpin-Tsai model [200], and the error was less than 4%. Taheri-Behrooz and Pourahmadi [201] analysed the heterogeneous RVE with the periodic boundary condition to predict the effective mechanical properties of the composites. The results of the periodic boundary condition were compared with the homogeneous boundary condition. In case of homogenous boundary conditions, the specified set of dependent variables and their derivatives are set to zero at the boundaries. While in the case of periodic boundary conditions, the displacements are continuous, meaning that the neighbouring RVEs cannot separate or overlap each other, and the tractions at the opposite parallel boundaries of an RVE must be the same. The study reported that homogeneous boundary conditions lead to an overestimate of RVEs’ effective mechanical properties. This is because of undesirable and over constrained homogenous boundary condition. The magnitude of 33% overestimate of homogenous boundary condition was highlighted in the literature. Tian, Qi [202] adopted the FE-based homogenisation method to evaluate the effective thermal conductivity of the composite materials. The thermal periodic boundary conditions were implemented with three different inclusions that is spherical, ellipsoidal and cylindrical. The proposed method was well agreed with Lewis-Nielsen model [203, 204].

The selection of an appropriate homogenisation strategy therefore depends on the required balance between computational efficiency and the level of microstructural detail necessary to capture process-induced material-property evolution.

### Transfer of process simulation to the structural level performance

Manufacturing-induced defects, such as fibre waviness and ply wrinkling, can be treated as sources of statistical variability that carry through to the structural failure probability [205]. It has been argued that current aerospace certification still relies partly on heuristic safety factors and an expensive multi-scale test programme because this process-to-performance link is not yet well established in a computational sense. To achieve a realistic assessment of composite structural performance, it is necessary to account for the component’s manufacturing history. Process simulations capture the development of curing kinetics, thermal gradients, and residual stress fields generated during fabrication. These process-induced states are often retained within the structure and can affect mechanical responses. Consequently, integrating process-simulation outputs into structural analyses yields a more representative prediction of component behaviour under service-loading conditions. The transfer procedure for implementing this involves extracting the final process-induced stress and deformation fields at each scale and applying them as initial conditions to the structural finite element model. This section explains how fibre orientation, thickness variation, residual stress, degree of cure or crystallinity, and porosity, all predicted by the process models earlier in this review, can in principle be mapped onto a structural finite element model as spatially varying material properties, and what this means for stiffness, strength, fatigue and damage tolerance assessment. This framework enables the direct assessment of the influence of manufacturing effects on the structural response.

**Fig. 13:**
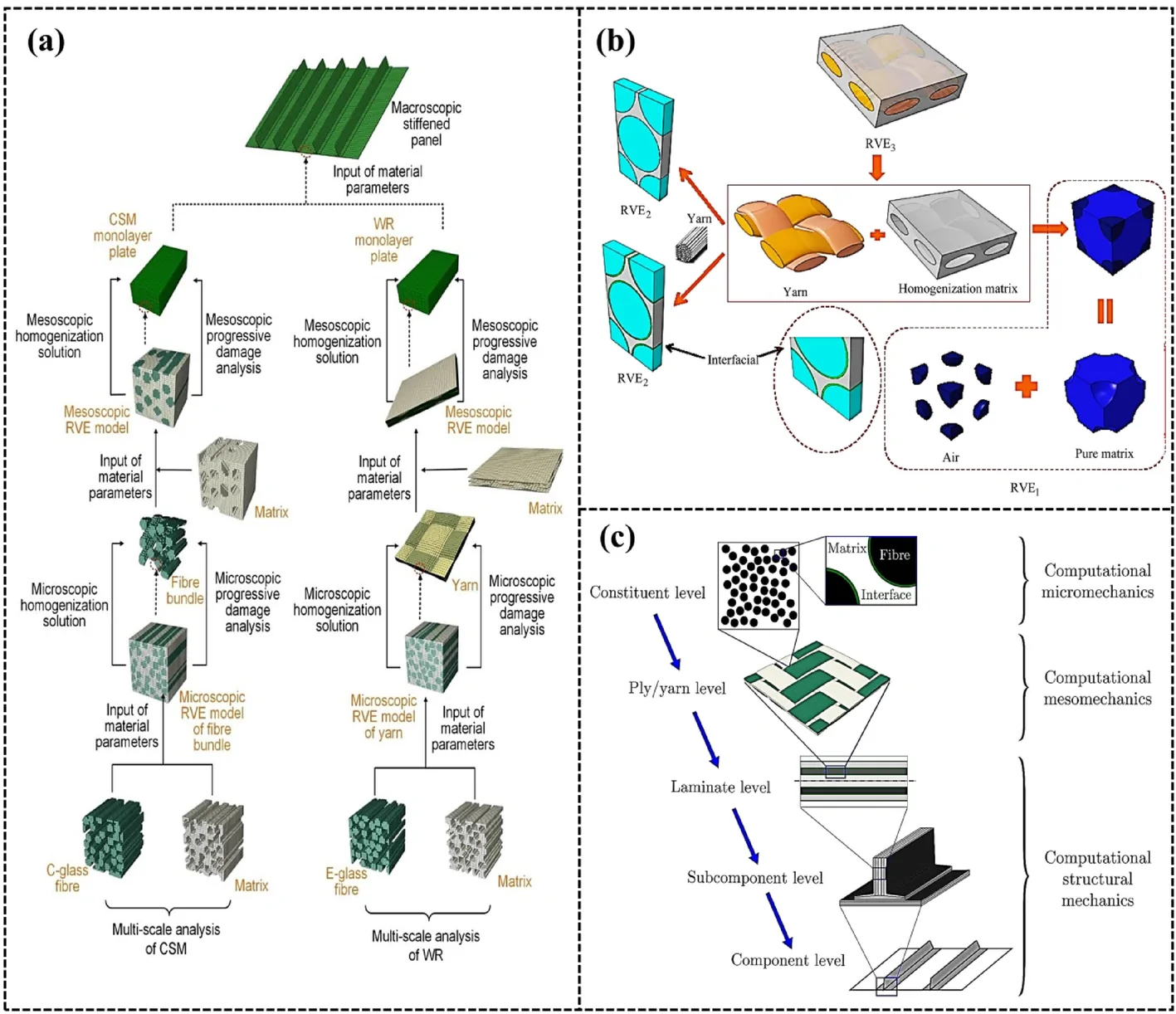
**a** Multiscale computational framework for predicting the mechanical response of stiffened composite panels [206], **b** Multiscale modelling approach applied to plain weave composite materials [207], **c** Hierarchical modelling strategy spanning from the constituent material level up to the structural component level [208]

Liu, Zhang [206] presented an analysis framework employing the multiscale method to examine the mechanical properties of the maritime GFRP stiffened panel. The framework applied an upscaling analysis approach, i.e. from micro to meso to macro. For chopped strand mat (CSM) plate, fibre bundles were at a mesoscale stage, and in woven roving (WR) composite fibre, woven yarn was at the mesoscale, as shown in Fig. 13a. A parametric study was conducted on a composite stiffened panel to determine the optimal ultimate strength by varying CSM parameters (volume fraction and fibre bundle diameter) and WR parameters (cross-sectional area of yarn and braided angle). It was observed that a 15% increase in the yarn’s cross-sectional area resulted in a corresponding 15% enhancement in the panel’s ultimate strength. Reducing the yarn braiding angle by approximately 20% resulted in an improvement of about 16% in the ultimate strength of the panel. In the case of CSM, an increase in fibre bundle content by 20% resulted in an approximately 5% enhancement in the final strength of the stiffened panel. The multiscale modelling of the plain weave composite to predict the thermal response considering the internal and interfacial defects was given by Zhao, Guo [207]. Representative volume elements (RVEs) were retrieved at different scales (micro-scale, mesoscale, and macro-scale) in order to accurately reflect the composite systems at each specific scale as shown in Fig. 13b. It was observed that the thermal conductivity of the composite was greatly affected by porosity and fibre volume fraction and was found to be decreased. Múgica, Lopes [208] established the multiscale modelling approach for glass fibre reinforced polypropylene woven composite to estimate the mechanical properties. The hierarchically based bottom-up strategy where from computational micromechanics (i.e. at the constituent level) to computational structural mechanics (i.e. component level) were employed as shown in Fig. 13c. Despite many advancements, uncertainty propagation remains a significant challenge in process-structure-performance frameworks, as uncertainties originating from material properties, boundary conditions and process parameters can accumulate across multiple simulation scales and influence final structural predictions.

### Other modelling methodologies

#### Meshless modelling

Conventional numerical discretisation techniques, including the Finite Element Method (FEM), Finite Volume Method (FVM), and Finite Difference Method (FDM), have been extensively utilised in composite process simulation to capture complex coupled physical phenomena. However, mesh generation for geometrically intricate components demands considerable expertise and iterative refinement, and despite significant advances in computational processing power, overall simulation turnaround time has not reduced proportionally [209]. Moreover, problems involving severe material deformation and propagating discontinuities require sophisticated numerical treatments that are fundamentally constrained by fixed mesh topology [210]. To overcome these limitations, meshless methods have emerged as a compelling alternative, eliminating structured domain discretisation and delivering reduced computational overhead alongside enhanced geometric modelling flexibility [211]. Smoothed Particle Hydrodynamics (SPH) [212], one of the most prominent mesh-free approaches, operates as a meshless Lagrangian framework in which the governing conservation equations are discretised using a moving particle assembly, each particle representing a discrete fluid volume and carrying associated physical state variables. SPH inherently accommodates exact advection, exhibits strong conservation properties, and demonstrates high adaptability in resolving large deformations, evolving interfaces, and free-surface dynamics. Since its original formulation, SPH has been progressively extended to dynamic structural response with material strength [213, 214], fracture mechanics [215], metal forming [215], simulate complex behaviour of viscoelastic fluids [216, 217], resin flow [218], 3D printing [218] and injection moulding simulation [210] or material processing simulation [219].

**Fig. 14:**
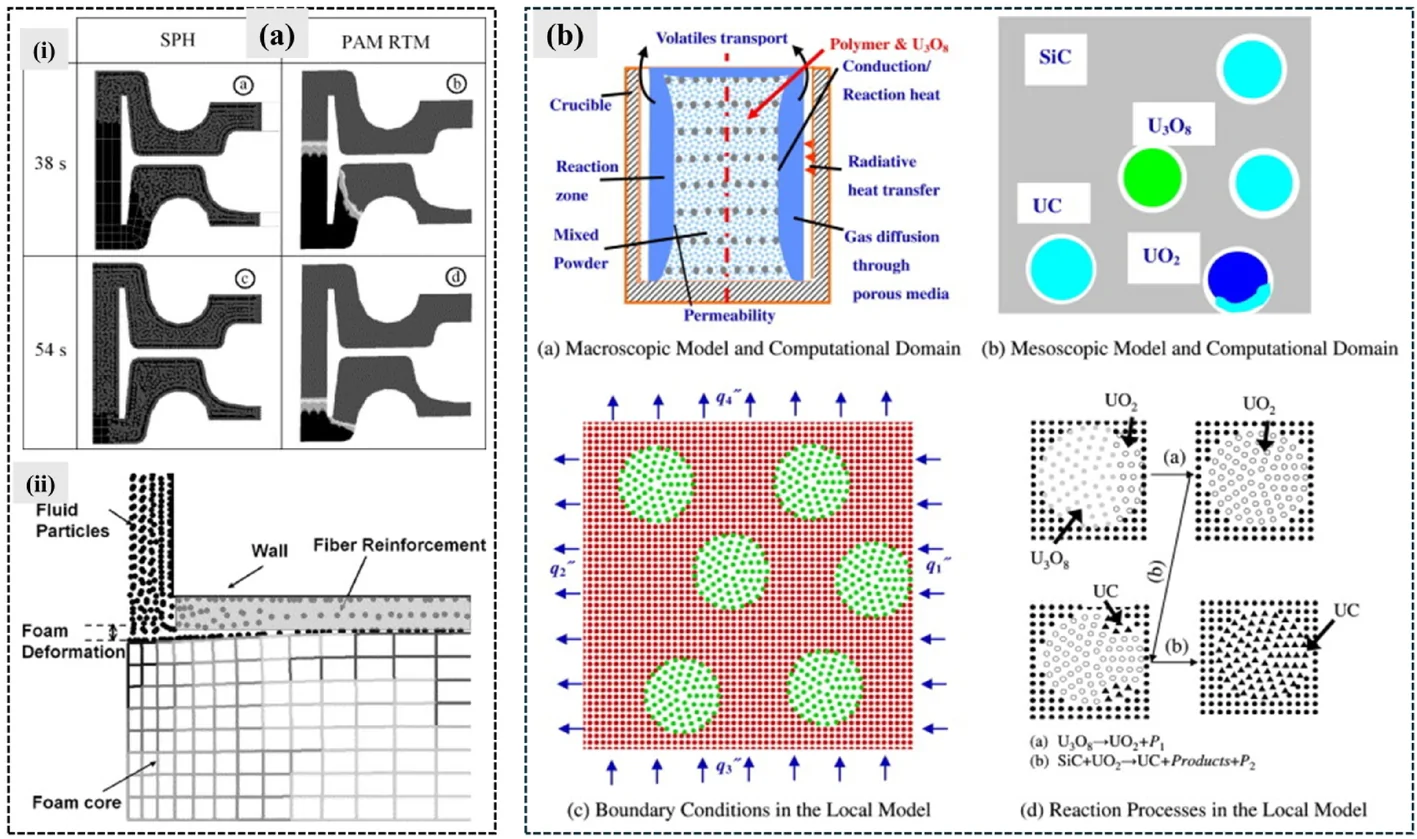
**a** (i) Flow front position comparison for isotropic permeability filling simulations, (ii) Part of the domain used for the FE/SPH simulation [218]; **b** The schematic of the SPH local model and its interaction with the global porous medium model [222]

Within the process simulation framework, the governing mathematical formulations underlying resin flow, viscous fluid dynamics, and injection moulding simulations are based on the mass conservation equation and the Navier-Stokes momentum equations. Comas-Cardona, Groenenboom [218] investigated Resin Transfer Moulding (RTM) of composite/foam-core sandwich structures through a coupled FEM-SPH methodology to address hydro-mechanical interactions, wherein SPH particles and Lagrangian finite elements were employed to represent the viscous resin phase and elastic foam core, respectively (Fig. 14a). The pressure-driven resin infiltration induced foam core deformation that deviated from initial boundary conditions due to permeability variations and the emergence of preferential flow channels. Hall, Qamar [220] extended SPH to the mending mechanism in self-healing composite systems, modelling capillary-driven resin infiltration into propagated cracks by incorporating surface tension as an externally applied body force within the SPH formulation. SPH has further been deployed to simulate fibre suspension flow in short steel fibre-reinforced concrete, treating the composite as a coupled system of a non-Newtonian incompressible fluid matrix and discrete rigid body inclusions [221], where individual fibres were represented by two-particle connected assemblies positioned at fibre endpoints, enabling real-time monitoring of fibre orientation and spatial distribution evolution throughout the moulding cycle. At the mesoscopic scale, an SPH-based process modelling framework was developed by Wang, Zhang [222] for ceramic matrix composite (CMC) fabrication, wherein solid-state reactions were governed by the continuity, energy, and species transport equations, and microstructural evolution was predicted as a function of heat flux. The reaction between SiC matrix and U₃O₈ filler particles produced continuous compositional gradients, porosity evolution, and property variations that were effectively captured through the inherently Lagrangian nature of SPH, which is particularly well-suited to tracking filler shrinkage and associated porosity redistribution (Fig. 14b). Huntley et al. [223] used the SPH method to simulate a discontinuous fibre composite manufacturing process. The process involves highly coupled fluid structure interactions, as the fibres are put in a water suspension and then sprayed onto a surface to create a discontinuous fibre tape where the fibres are aligned in one direction. The developed SPH models model the process well, and the process parameters affecting fibre alignment can be captured. Though meshless approaches offer particular advantages for simulations involving large deformations, moving boundaries and evolving free surfaces, where traditional finite-element meshes can become highly distorted. However, challenges associated with computational cost, enforcement of boundary conditions and industrial implementation have limited their widespread adoption in composite-manufacturing simulations.

While SPH demonstrates considerable versatility in addressing coupled multiphysics problems, SPH carries several significant computational and methodological limitations in the context of composite process modelling. The computational cost of fine-particle discretisation required to resolve microscopic composite microstructures becomes prohibitively high as the total particle count increases to realistically represent macroscopic target geometries. Compounding this, the repeated updating of neighbouring particle lists is an inherently time-intensive algorithmic operation that must be executed at frequent intervals, further amplifying the overall computational burden.

#### Machine learning (ML)-based process modelling

**Fig. 15:**
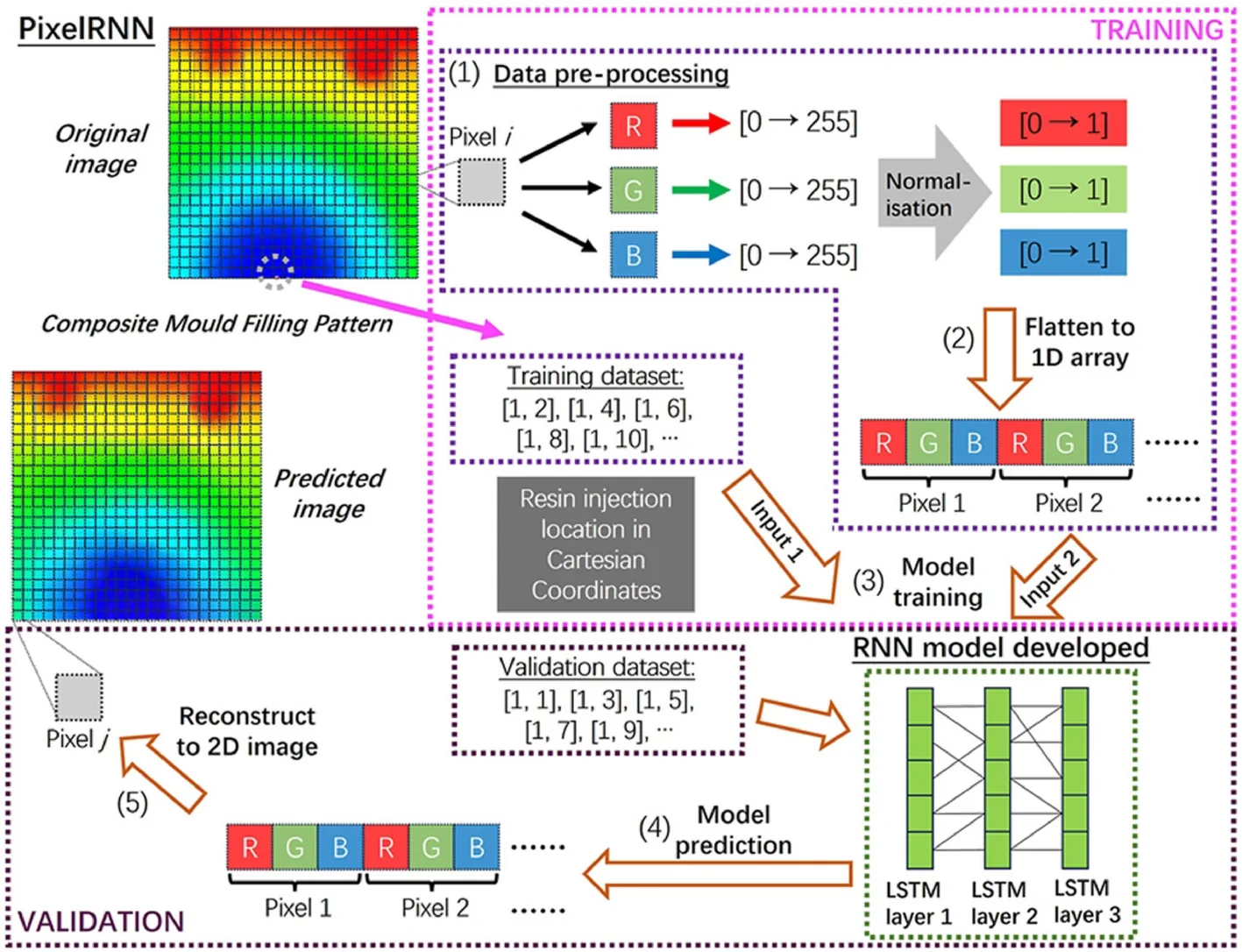
Schematic diagram depicting the operations of the PixelRNN during model training and validation [230]

While the application of machine learning (ML) methods in the field of composite processing modelling is still in its nascent stages, it has been increasingly gaining traction in recent literatures [224]. Cassola, Duhovic [225] presented a good review on the research works already exploiting ML methods for polymer composite process simulation, categorised by manufacturing technologies in five categories: (1) forming of fibre reinforcement structures, (2) flow in fibre reinforcement structures, (3) fibre filled moulding compound flow, (4) automated fibre placement/winding and (5) autoclave processing. For example, Pfrommer, Zimmerling [226] considered the forming of a rectangular non-crimp fabric into the shape of an automotive door reinforcement beam. In the process, the fabric was fixed by 50 grippers, attached to a surrounding frame by springs. ANNs were then used to create a surrogate model for the optimisation of the process parameters. Stieber, Schröter [227] constructed a CNN-based binary classifier, named FlowFrontNet, to predict dry spots in preforms during RTM processes. The data for model training was acquired from 40,000 simulations using PAM-RTM from which 6 million images were extracted and automatically labelled using the Python library OpenCV. Carlone, Aleksendrić [228] addressed the autoclave heat convection problem by modelling cure cycles (temperature over time) using Recurrent Neural Networks (RNN). Humfeld, Gu [229] aimed to control the autoclave air temperature by deploying MultiLayer Perceptrons (MLP)- and Long Short-Term Memory (LSTM)-models in order to optimise the cure cycle for simultaneously processed aerospace parts. Apart from the above, Goli, Vyas [224] developed a Deep Neural Network named “ChemNet” to predict and optimise the cure kinetics of the matrix phase of the thermoset composites for a specific application (a desired volume fraction of fibres) and a favourable manufacturing strategy (expected maximum temperature etc.) via the frontal polymerisation process. Wang, Xu [230] utilised PixelRNN, an image-based machine learning model, to predict the mould filling pattern of the RTM process, as shown in Fig. 15 .

Unlike traditional physics-based modelling approaches that rely heavily on fundamental theoretical frameworks, these ML-based techniques are primarily characterised as theory-agnostic (or data-driven) methodologies. This means that they do not require prior knowledge of the underlying physical mechanisms, chemical reactions, or material behaviours that govern composite processing . The main step in these approaches is to create a dataset of inputs (e.g. process parameters) and desired outputs. The dataset should be large enough to allow for successful training of ML models such as Neural Networks (NN). In these applications, NNs are basically complex and sophisticated data fitting algorithms. The current ML-based approaches in composite processing modelling face two prominent challenges. The first major hurdle is the acquisition of large-scale, high-fidelity physical datasets. Generating such datasets often involves extensive experimental trials or high-fidelity simulations (e.g., finite element (FE) analysis), both of which are time-consuming, resource-intensive and costly, especially for composite materials, which exhibit significant variability in properties due to raw material differences and processing fluctuations. The second key limitation is the phenomenon known as “brittle AI”, which refers to the poor generalisation ability of NNs outside their training data distribution. In other words, if the model is presented with new input data that falls outside the range or characteristics of the data used for training (e.g., untested processing parameter combinations or novel composite material systems), the NN’s predictions may become inaccurate, unreliable, or even completely invalid.

Nevertheless, ML models hold a distinct advantage over traditional FE models in terms of computational efficiency. FE models, while highly accurate for physics-based simulations, often require substantial computational resources and long runtimes, particularly for complex composite processing scenarios involving multi-physics coupling (e.g., heat transfer, fluid flow, and mechanical deformation). In contrast, once trained, ML models can generate predictions in a matter of milliseconds or seconds, making them highly suitable for developing real-time predictive tools. Such tools have significant practical value in industrial settings, as they can enable on-the-fly adjustment of processing parameters, real-time monitoring of part quality, and rapid optimisation of composite manufacturing processes, thereby improving production efficiency, reducing defect rates, and lowering overall manufacturing costs. Despite their growing potential, ML models remain dependent on the quality, quantity and representativeness of the available training data. Models trained on a specific material system, geometry or processing window may exhibit limited generalisation when applied outside the calibration domain. Furthermore, purely data-driven approaches often lack physical interpretability, making extrapolation to novel manufacturing conditions challenging. For this reason, increasing attention is being directed towards hybrid approaches that combine ML with physics-based process models, allowing data efficiency and computational speed to be improved while maintaining physical consistency.

####  Reduced-order, PINNs and digital twins models and short summary

Beyond the data-driven surrogate models already discussed [224–230], two related categories deserve explicit treatment. Reduced-order models (ROM) construct a lower-dimensional response surface or regression fit from a limited set of full-order FE simulations, trading some accuracy for evaluation speeds fast enough to embed inside an optimisation loop; this approach has already been applied to reduce VARTM simulation cost [79, 91]. Physics-informed neural networks (PINNs) sit in a middle ground between pure data-driven ML and full physics-based FE, rather than fitting to simulation or experimental output data alone, a PINN trained by embedding the governing differential equations directly into the loss function, so the network is constrained to satisfy the underlying physics, such as the coupled heat transfer and cure kinetics equations, even where training data is sparse. A demonstrated example solves the coupled thermochemical curing problem for a composite-tool system using two disconnected subnetworks trained sequentially to handle the differing timescales of thermal conduction and resin cure [231]. This class of model is particularly relevant where full FE runs are too expensive to embed in real-time process control, but pure data-driven surrogates would need more training data than is practically available.

A digital twin extends surrogate and reduced-order modelling (ROM) from offline prediction to real-time coupling with process-sensor data, updating the simulation as the manufacturing process progresses rather than relying solely on pre-process predictions. A demonstrated example in resin transfer moulding (RTM) pairs a small array of in-mould pressure sensors with two deep-learning surrogate sub-models: one detects flow disturbances such as race-tracking, while the other predicts the resulting flow-front evolution and pressure field. Both models were trained on synthetic data generated from full-order flow simulations and validated against experimental injection data [232]. This represents one of the clearest demonstrations to date of closing the loop between process simulation and real-time manufacturing control. However, its current scope is limited to the detection of a single defect mechanism (race-tracking) within a single manufacturing process (RTM), and the extension of similar approaches to cure-induced residual stresses, distortion prediction and thermoplastic consolidation defects remains an open research challenge.

Full thermochemical and thermomechanical FE simulations remain the most accurate approach for predicting cure, flow and residual stress development, but their computational cost can limit their application in optimisation and real-time control [10]. Consequently, reduced-order models and surrogate approaches are increasingly employed to provide rapid predictions [79, 91]. PINN and digital-twin approaches occupy an intermediate position by combining physical constraints with improved computational efficiency [231, 232]. In practice, the preferred modelling strategy considers the balance between required accuracy, computational cost and industrial application needs. The suitability of each modelling strategy depends on the manufacturing scenario being considered. Finite element methods remain the preferred choice for process-scale thermal, mechanical and residual stress analyses due to their maturity and industrial adoption. Molecular dynamics is most valuable where atomistic mechanisms, constituent behaviour or interfacial properties are required, although its computational cost limits application to small length scales. RVE-based multiscale approaches are advantageous when process-induced microstructural features must be linked to structural performance. Meshless methods such as SPH are particularly useful for problems involving large deformation, free-surface flow or evolving interfaces, including mould filling and resin flow. Machine-learning approaches are most attractive for optimisation, process control and rapid prediction once sufficient training data are available. Consequently, no single strategy is universally optimal, and the choice should be guided by the dominant physical mechanisms, required accuracy and available computational resources.

The modelling approaches reviewed in this section span a wide range of length scales, from molecular dynamics at the atomistic level [160–162] to ML-based surrogate models operating directly at the process scale [224–230]. RVE-derived effective properties in the studies reviewed here are computed once and passed to a separate structural-scale model without further feedback [180–187, 189–191, 193, 194, 197, 199, 201, 202]; whether this decoupled approach or the increasingly direct ML-based surrogate route proves more durable as computational cost continues to fall is not yet resolved by the literature covered in this review.

Table 2 compares the different modelling approaches used in composite manufacturing across different length and time scales, computational requirements, industrial maturity and application domains. The comparison highlights the trade-offs between model fidelity, computational efficiency and practical applicability, ranging from molecular-scale simulations to real-time digital twin frameworks.

**Table 2:** Comparison of different modelling approaches of composite processing

Approach	Typical length/time scale	Computational cost	Industrial readiness	Typical application	Major limitation
Molecular dynamics [160–162]	nm/ns (General MD characteristic)	Very high	Research only	Informing continuum model parameters (interfacial properties, deformation mechanisms)	Timescale limited to nanoseconds; cannot simulate an actual process step directly
RVE-based homogenisation [180–187, 189–191, 193, 194, 197, 199, 201, 202]	µm-mm	Medium	Established	Extracting effective (homogenised) CTE, modulus and strength for a separate structural-scale FE model	Local stress concentration details may be lost; homogenisation is decoupled (computed once), not concurrent with the macro solve
Conventional FEM [10, 49]	mm-m/s-h	Medium–Very high	Established	Curing, consolidation, thermal behaviour, residual stress, distortion and structural response	Computationally expensive for highly coupled, multiscale or large-scale problems
Meshless/SPH [209–214]	mm	Low relative to FEM in general [211], but rises sharply with particle count for fine microstructure resolution	Emerging in industry	Large-deformation/free-surface problems: resin infiltration, fibre suspension flow, self-healing crack infiltration, discontinuous fibre spraying	Fine-particle discretisation cost and neighbour-list updating overhead become prohibitively high as particle count increases
Data-driven ML/surrogate modelling [224–230]	Dependent (data-driven)	Low after training, high time consuming to generate training data	Emerging	Fast prediction of process outcomes once trained	Accuracy depends strongly on training data coverage; poor extrapolation and limited physical interpretability
Physics-informed neural networks (PINN) [231]	Dependent (physics-constrained)	Medium (training), very low at inference	Early-stage/research	Solving coupled thermochemical equations with sparse data	Training stability issues from differing physical timescales
Digital twin [232]	Structural/process scale, real-time	Low at run-time (~ 38ms/query)	Early-stage/demonstrated in composite curing and food manufacturing	Real-time correction of a manufacturing run against sensor data	Relies on offline-trained AI; no embedded real-time simulation

Predictive capability is not reported consistently across the models reviewed here and is best considered across three distinct dimensions: calibration accuracy, validation accuracy and the generalisation capability. The calibration accuracy refers to the ability of a model to reproduce the experimental dataset it was fitted to. In the studies reviewed, calibration is typically performed by fitting directly to DSC, DMA, XRD data or other material characterisation data, however, standalone goodness-of-fit measures are not consistently reported. The validation accuracy refers to the performance of a model against independent experimental, analytical or benchmark data that were not used for model calibration. This is reported inconsistently across the literature, nevertheless, where reported, these results suggest that accuracy varies more strongly by model category than by material system. For example, RVE-based homogenisation methods have reported errors below 4% when compared against the Halpin-Tsai analytical benchmark [199]. The autoclave thermal field predictions are reported at a deviation of 5.9% from experiment measurements using a computational fluid dynamics approach [118], while a coupled quasi-transient CFD–FE strategy reduced the reported deviation to 2.3% (4.9 °C) [, 119, 120]. The generalisation capability is defined as the ability of a model to perform outside the conditions under which a model was trained or calibrated. Among the three dimensions, generalisation is the least well-demonstrated across the models reviewed. This limitation is particularly evident in machine-learning-based approaches, where predictive performance can decrease when new conditions fall outside the training-data distribution. However, generalisation is also insufficiently assessed for many physics-based constitutive models, where parameters calibrated for a particular material or process condition are often applied to different conditions without independent transferability studies. Consequently, future studies should report not only calibration and validation accuracy but also model performance under changes in material properties, processing conditions, geometry and boundary conditions.

Across the modelling approaches reviewed in this article Sect. 2-4, a clear trade-off exists between predictive fidelity, required material characterisation and computational cost. At the constitutive level, the Kamal-Sourour autocatalytic model [4] requires only DSC-derived kinetic parameters and is computationally efficient, but its assumption of a single dominant reaction mechanism may limit applicability for more complex resin systems. Similarly, the CHILE approach [33] achieves computational efficiency by representing modulus evolution through cure-dependent property development rather than a full viscoelastic formulation and therefore remains widely adopted in industrial process simulations. In contrast, Generalised Maxwell-Prony viscoelastic models [56] provide a more realistic representation of stress relaxation and time-dependent material behaviour but require extensive DMA characterisation and greater computational effort. At the process-simulation level, molecular dynamics can capture atomistic mechanisms inaccessible to continuum-scale approaches but is restricted to nanometre length scales and nanosecond timescales, making it more suitable for informing constitutive parameters than directly modelling industrial manufacturing processes [160–162]. RVE-based homogenisation efficiently transfers microscale information to structural-scale simulations, although local stress concentrations may not be fully resolved [180–185, 188–190, 199–202]. Meshless methods such as SPH offer advantages for problems involving large deformations, evolving interfaces and free-surface flows but usually require higher computational resources than conventional finite-element approaches [209–211]. Machine-learning and reduced-order approaches provide significant computational speed advantages once trained; however, their predictive capability depends strongly on the quality and representativeness of the training data. Consequently, no single modelling methodology is universally optimal, and the selection of an appropriate framework depends on the balance between required accuracy, computational efficiency, available characterisation data and industrial application requirements.

While many of the modelling approaches reviewed have demonstrated strong predictive capabilities under controlled laboratory conditions, their robustness in industrial manufacturing environments remains a significant challenge. Industrial composite manufacturing is characterised by unavoidable variability arising from batch-to-batch differences in raw materials, fibre placement inaccuracies, tooling tolerances, thermal gradients, machine calibration, and environmental fluctuations. Most physics-based models rely on deterministic material properties and nominal process parameters, which may not fully capture the associated uncertainty. Consequently, prediction accuracy can degrade when models are transferred from laboratory-scale validation to production-scale applications. Recent research has therefore emphasised uncertainty quantification, stochastic modelling, process monitoring, and digital twin frameworks to improve model reliability under realistic manufacturing conditions. Integrating real-time sensor data with machine learning-based model updating offers a promising pathway to enhance robustness and facilitate deployment in industrial composite manufacturing environments.

## Conclusion

The processing of fibre-reinforced polymer composites is governed by strongly coupled multiphysics phenomena, where interactions among flow behaviour, thermal transport, phase transformation, and mechanical response collectively to determine the final material state and the structural performance of composite components. The inevitable development of residual stresses during manufacturing remains a critical challenge, as their non-uniform distribution can induce defects such as matrix cracking and delamination, as well as macroscopic distortions that compromise dimensional accuracy. This review has synthesised current advances in process modelling and numerical simulations, with particular emphasis on thermokinetic, chemorheological, and flow-related formulations, as well as thermomechanical frameworks for both thermoset and thermoplastic systems. Constitutive descriptions capturing evolving material behaviour under processing conditions have also been examined, highlighting their pivotal role in accurately predicting stress evolution and process-induced deformation. Furthermore, the importance of multiscale approaches has been underscored, as they enable the linkage between microscale mechanisms and macroscale structural response, thereby improving the fidelity of process simulations.

Despite significant progress, challenges remain in achieving fully predictive, computationally efficient models that robustly account for the complexity of coupled phenomena and real-world process variability (e.g., inconsistent material properties, variable processing parameters). The gaps identified throughout this review can be grouped into several recurring themes, rather than being considered as isolated issues. First, model coupling remains incomplete. Although individual approaches for flow, thermal, and mechanical fields, as well as multiscale approaches linking the RVE to the structural scale, have been individually well developed, they are rarely integrated into a single, fully validated process-to-structure workflow. Second, predictive confidence remains limited by insufficient uncertainty assessment and model transferability. Uncertainty quantification is rarely reported in composite process modelling studies, despite its established role in adjacent fields such as structural reliability. The lack of demonstrated transferability of calibrated constitutive parameters across polymer systems means that each new material system currently requires re-characterisation from first principles rather than effective reuse of prior calibration data. Third, several defect-generating mechanisms, porosity evolution, fibre waviness, and void transport, are acknowledged qualitatively across the literature studies but are often treated separately or qualitatively rather than being fully coupled with the flow, cure, or crystallisation models. Fourth, computational cost remains an important practical barrier preventing high-fidelity models from being used routinely in industrial design loops rather than only in research settings. The large number of material and process variables, together with their inherent variability, further increases the difficulty of developing robust and computationally efficient models [233]. Emerging approaches reviewed in this article, including physics-informed neural networks, digital-twin implementations, and data-driven optimisation strategies, offer promising opportunities to reduce computational burden while retaining physical fidelity [234], represent the most promising current routes to closing this gap. However, their applicability, robustness and validation across different composite manufacturing processes and material systems remain to be demonstrated.

Future developments are expected to focus on integrated multiscale process-structure-performance frameworks that bridge atomistic, mesoscopic and continuum scales while incorporating uncertainty quantification to account for material variability and process fluctuations. Increasing attention is being directed towards hybrid modelling approaches combining finite element analysis with machine learning, reduced-order models and physics-informed neural networks to improve predictive capability while reducing computational cost. Furthermore, the emergence of digital-twin technologies, integrated process monitoring and data-driven model updating offers significant potential for real-time manufacturing optimisation, defect mitigation and closed-loop process control. The availability of standardised benchmark datasets and improved model interpretability will also be essential for accelerating industrial adoption and strengthening confidence in advanced process-modelling methodologies.

It is also important to emphasise that the unified modelling framework discussed throughout this review should not be interpreted as a single constitutive model or numerical method capable of describing all composite manufacturing processes. Instead, a unified framework is more appropriately viewed as a modular process-structure-performance architecture in which different modelling approaches operate at the scales and physics for which they are most suitable. Finite element methods remain the preferred tools for continuum-scale thermal, mechanical and deformation analyses; molecular dynamics provides atomistic-level insights into constituent and interfacial behaviour; meshless methods are advantageous for problems involving severe deformation and evolving interfaces. Meanwhile machine-learning and reduced-order models offer rapid prediction capabilities for optimisation and process control. Within such a framework, process-specific models are coupled through the transfer of information across scales rather than being replaced by a single universal methodology. A fully universal modelling framework capable of accurately representing all composite manufacturing processes remains unlikely because of the wide range of material systems, processing routes, length scales, timescales and constitutive behaviours involved. Consequently, future developments are likely to favour interoperability between specialised models rather than a single all-encompassing modelling approach.

Despite substantial advances in constitutive modelling, process simulation and multiscale analysis, the principal challenge remains the integration of multiphysics, multiscale and data-driven methodologies within a single validated framework. Future progress will depend not only on improvements in individual modelling techniques but also on their effective coupling through robust process-structure-performance strategies. The successful integration of high-fidelity physics-based simulations, uncertainty quantification, machine learning and digital-twin technologies has the potential to transform composite manufacturing by enabling more accurate, efficient and industrially deployable predictive tools for next-generation composite structures.

## Data Availability

No datasets were generated or analysed during the current study.
